# Repair of traumatic lesions to the plasmalemma of neurons and other cells: Commonalities, conflicts, and controversies

**DOI:** 10.3389/fphys.2023.1114779

**Published:** 2023-03-15

**Authors:** Marshal L. Mencel, George D. Bittner

**Affiliations:** ^1^ Institute of Cell and Molecular Biology, University of Texas at Austin, Austin, TX, United States; ^2^ Department of Neuroscience, University of Texas at Austin, Austin, TX, United States

**Keywords:** plasmalemmal/axolemmal disruptions, axonal severance, apoptosis, membrane repair, polyethylene glycol, calcium

## Abstract

Neuroscientists and Cell Biologists have known for many decades that eukaryotic cells, including neurons, are surrounded by a plasmalemma/axolemma consisting of a phospholipid bilayer that regulates trans-membrane diffusion of ions (including calcium) and other substances. Cells often incur plasmalemmal damage *via* traumatic injury and various diseases. If the damaged plasmalemma is not rapidly repaired within minutes, activation of apoptotic pathways by calcium influx often results in cell death. We review publications reporting what is less-well known (and not yet covered in neuroscience or cell biology textbooks): that calcium influx at the lesion sites ranging from small nm-sized holes to complete axonal transection activates parallel biochemical pathways that induce vesicles/membrane-bound structures to migrate and interact to restore original barrier properties and eventual reestablishment of the plasmalemma. We assess the reliability of, and problems with, various measures (e.g*.*, membrane voltage, input resistance, current flow, tracer dyes, confocal microscopy, transmission and scanning electron microscopy) used individually and in combination to assess plasmalemmal sealing in various cell types (e.g., invertebrate giant axons, oocytes, hippocampal and other mammalian neurons). We identify controversies such as plug *versus* patch hypotheses that attempt to account for currently available data on the subcellular mechanisms of plasmalemmal repair/sealing. We describe current research gaps and potential future developments, such as much more extensive correlations of biochemical/biophysical measures with sub-cellular micromorphology. We compare and contrast naturally occurring sealing with recently-discovered artificially-induced plasmalemmal sealing by polyethylene glycol (PEG) that bypasses all natural pathways for membrane repair. We assess other recent developments such as adaptive membrane responses in neighboring cells following injury to an adjacent cell. Finally, we speculate how a better understanding of the mechanisms involved in natural and artificial plasmalemmal sealing is needed to develop better clinical treatments for muscular dystrophies, stroke and other ischemic conditions, and various cancers.

## 1 Introduction to plasmalemmal sealing

### 1.1 A brief history of plasma membrane repair

Eukaryotic cells in multicellular organisms often incur damage to their plasmalemma, the membranous diffusion barrier between the extracellular and intracellular fluids that completely surrounds each individual cell (Singer and Nicolson, 1972; Leonard, 1993; Alberts et al., 2015). The plasmalemma typically consists of a phospholipid bilayer studded with proteins, as do other membrane-bound organelles. Water molecules bound to the inner and outer surface of hydrophilic phospholipids and proteins of the plasmalemma and other membranous vesicles/organelles prevent their spontaneous fusion. For example, membrane-bound water prevents the plasmalemma of closely apposed cells in a tissue from spontaneously fusing, thereby preventing tissues composed of individual cells from forming a multinucleate cytoplasmic syncytium with no plasmalemmal boundaries. Membrane-bound water also prevents intracellular vesicles and organelles from readily fusing with each other and/or the plasmalemma (Singer and Nicolson, 1972; Alberts et al., 2015).

**FIGURE 1:**
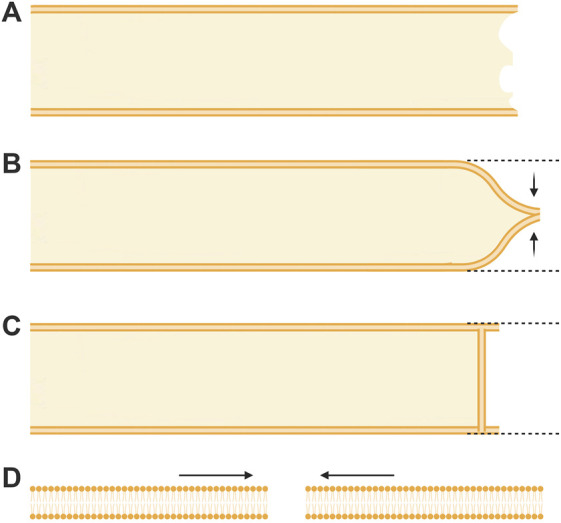
Topographical models of previously assumed mechanisms of axonal sealing. **(A)** An axon completely transected on the far right prior to any repair processes. **(B)** Sealing of a transected axon by collapse and fusion of apposed plasmalemmal leaflets. **(C)** Sealing of a transected axon by spontaneous formation of a membranous partition-like structure. **(D)** Sealing of a very small (nm) membrane disruption by the thermodynamically favorable lateral migration of lipids.

Traumatic damage to a plasmalemma ranges from a small hole of nanometer-to micron-sized diameters to complete transections of a cell body or its cytoplasmic extensions (i.e., filopodia, dendrites, axons, etc.) that may be many (2–1,000) microns in diameter (LaPlaca, 2009; Vargas and Bittner, 2019; Kang et al., 2020). Prior to 1994, if covered at all in textbooks, repair of a complete transection of an intact cytoplasmic extension (Figure 1A) was assumed to occur by collapse and fusion of the apposing plasmalemmal leaflets at each cut end (Figure 1B) or the formation of a membranous partition (Figure 1C) (Yawo and Kuno, 1983; Yawo and Kuno, 1985; Kelly, 1985, 1991). A smaller hole anywhere in the plasmalemma was assumed to repair by the spread and fusion of lipid bilayers surrounding the hole (Figure 1D) (reviewed in McNeil and Baker, 2001; Horn and Jaiswal, 2019). Such repairs were assumed to take microseconds to milliseconds to completely seal the damaged plasmalemma, i.e., to restore diffusion properties of an intact plasmalemmal barrier, especially to prevent the influx of extracellular calcium (Schlaepfer and Bunge, 1973; Krause et al., 1994; reviewed by Vargas and Bittner, 2019).

**FIGURE 2:**
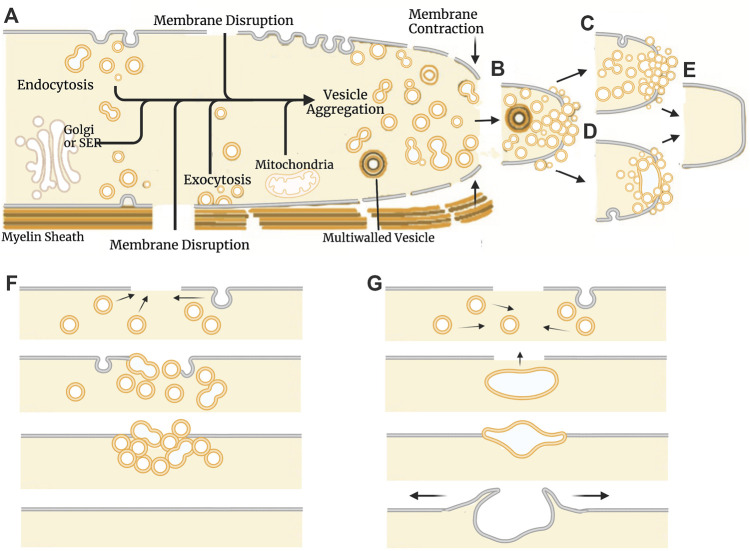
Models of natural plasmalemmal repair mechanisms. **(A)** Cellular components and events involved in vesicle-mediated membrane repair. Influx of Ca^2+^ through membrane disruptions leads to the formation of membranous structures that aggregate at the site of injury. The Golgi apparatus, smooth endoplasmic reticulum (SER) and mitochondria can all contribute to vesicle formation and subsequent repair processes. Vesicles can arise from any membranous source including the plasmalemma, organelles, and surrounding glia/myelin. Vesicles continue to aggregate at the site of injury **(B)** and form functional barrier either through a plug **(C)** or patch **(D)** mechanism. Regardless of mechanism, repaired cells eventually form a continuous bilayer at the site of previous injury **(E)**. Schematic representation of the events described by the plug **(F)** and patch **(G)** hypothetical mechanisms. The plug hypothesis features progressive formation of a diffusion barrier by dense packing of vesicles followed by formation of a continuous plasmalemma. The patch hypothesis involves formation of a large wound vesicle that seals in a single-step fusion event followed by the collapse of this structure (explodosis). The outer plasmalemma is shown in gray while intracellular membrane-bound components (i.e., vesicles, organelles, etc.) and endocytotic vesicles are shown in orange. Adapted from Vargas and Bittner (2019).

**FIGURE 3:**
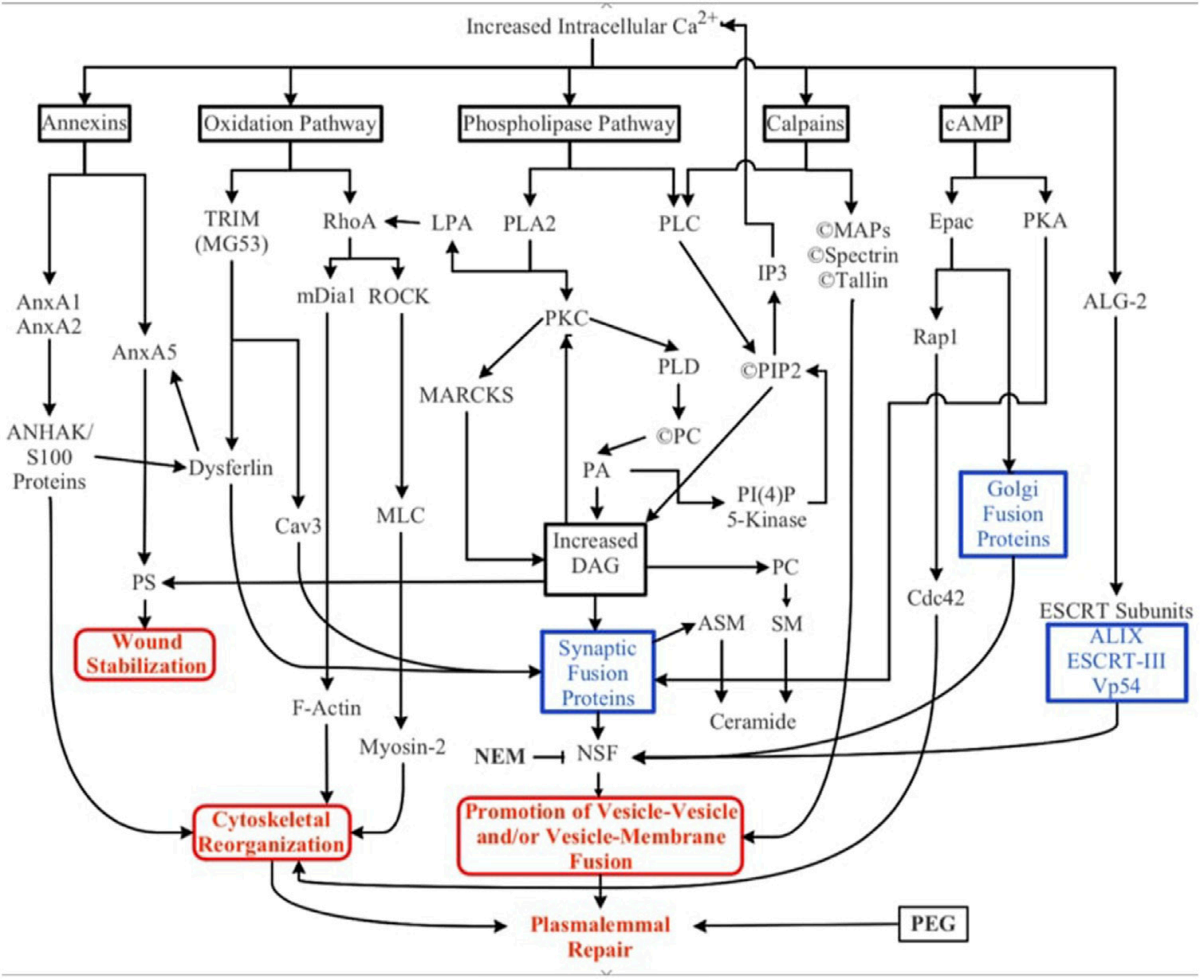
Biochemical pathways involved in natural and artificial repair of plasmalemmal disruptions. Membrane disruption allows an influx of Ca^2+^ into the cell due to the transmembrane concentration gradient of Ca^2+^. Increased intracellular Ca^2+^ activates several parallel pathways involving annexins, oxidative responses, phospholipases, calpains (and other proteases), cAMP, and dynamic changes to membrane lipid compositions. ESCRT proteins may participate in the closure of small nm‐sized holes that may involve membrane externalizations. Solid black lines and boxes outlined in black describe general pathways. Boxes outlined in blue describe the involvement of multiple cytoplasmic substances. Boxes outlined in red describe sub-cellular biophysical/morphological events. All these naturally-occurring pathways, cytoplasmic substances, and subcellular organelles converge onto three cellular biophysical/morphological events common to membrane sealing/repair in all eukaryotic cells: stabilization of the membrane surrounding the disruption, reorganization of cytoskeletal elements, and promotion of vesicle-vesicle and/or vesicle-membrane interactions. Finally, polyethylene glycol (PEG) is an artificial fusogen that bypasses all known Ca^2+^-dependent natural sealing pathways to rapidly repair plasmalemmal disruptions (PEG-sealing).

In 1994, two laboratories (Krause et al., 1994; Steinhart et al., 1994), working without the knowledge of the other’s research efforts, independently reported that cells seal holes in, or complete transections of, a eukaryotic plasmalemma in oocytes, axons, or epithelial cells by an accumulation, migration, and fusion of vesicles and other membranous structures (Figure 2). These and subsequent studies by various laboratories (reviewed in part by Moe et al., 2015; Vargas and Bittner, 2019) show that many (perhaps all) eukaryotic cells exhibit an influx of extracellular calcium (Ca^2+^) at the wound site that is required to initiate sealing. This influx of extracellular Ca^2+^ activates parallel biochemical pathways, intracellular organelles common to vesicular trafficking in the Golgi apparatus, conventional exocytosis, and coordinated exocytosis at chemical-releasing synapses (Figures 2, 3).

These repair processes shared by all eukaryotic cells (partially reviewed by Vargas and Bittner, 2019) have some conflicting details as reported for different cell types (e.g., oocytes vs. neurons vs. epithelial cells) and several controversies on specific sealing mechanisms (*e.g.*, plug vs patch, vesicle shedding vs. lipid spreading). That is, sealing dynamics by vesicular interactions that occur after onset of plasmalemmal trauma take seconds to minutes in different cell types to restore a plasmalemmal seal having diffusion barrier properties observed for their originally-intact plasmalemma. These vesicular interactions could form a vesicular “*plug*” that produces a constantly changing diffusion barrier that gradually attains the barrier properties of the intact plasmalemma (Figures 2A–C, E, F; Bittner et al., 2016b). Alternatively, vesicles could interact to form a large wound vesicle that immediately seals the membrane as a “*patch*” in one final step to attain the diffusion properties of an intact membrane (Figures 2A, B, D, E, G; Andrews et al., 2014; Davenport et al., 2016). Furthermore, several studies have also reported that amphoteric substances like polyethylene glycol (PEG) can restore a plasmalemmal barrier exceedingly rapidly, perhaps within microseconds to milliseconds *via* a mechanism similar to original hypotheses for sealing plasmalemmal transections by direct fusion of plasmalemmal leaflets (Figure 1B) or holes by lipid spreading (Figure 1D) (Spaeth et al., 2012b; Zuzek et al., 2012; Vargas and Bittner, 2019).

### 1.2 Structure of this review

In this review, we summarize data on initial, intermediate, and final steps of plasmalemmal sealing to begin to correlate cellular/molecular with biochemical/biophysical events that are common to sealing in all eukaryotic cells. We also identify data that describe conflicting specific properties of different eukaryotic cell types and describe controversies regarding how current data on plasmalemmal sealing should be interpreted (Summarized in Table 1).

**TABLE 1:** Methods of assessing membrane repair.

	Invertebrate GAs	Oocytes	Mammalian neurites	Mammalian epithelial cells	Muscle cells
Electrophys-iological (R_in_, V_m_, I_i_)	5,6,7,9,10	–––––––––	19,20,23	–––––––––	–––––––––
Fluorescence	5,6,7,8,10	16,17,18	19,24,27,28,30	31,32,35^H^,40,4^H^,44^H^,47,49,51	55,59,60,61^H^,65
Confocal	1,3,4,5,6,9,11	14,17,18	20,22,23,29	31,33,35^H^,36,37,39,43^H^,45,46,49,50^H^,51,52,53	55,56,58,65^H^,66^H^
Immunostaining	3	14,16	22	37,40,43^H^,44^H^,47	55,56,58,61^H^,63^H^,64^H^,65,66^H^
SEM	2	15	–––––––––	33	–––––––––
TEM	1,4,6,8,9,10,11	17	20	32,33,35,37,43^H^,46,49	57^H^,58,66^H^

Table 1. **Methods of assessing the state of a membrane seal.** Numbers in the table represent original research articles that have used these methods to assess membrane repair in the various model systems (see Supplementary Materials for corresponding list of references). Note that several model systems have not been assessed by all available methods (especially by electrophysiological or scanning electron microscopy (SEM)) or have been assessed using certain methods in only one publication. A superscript H (X^H^) indicates that the referenced paper used human cells as a model system.

This review first assesses the general appropriateness, specific applications, and controversies of different techniques and measures used to assess the state of a plasmalemmal seal (Section 2), followed by a brief description of the model systems primarily used to study sealing after traumatic membrane damage (Section 3). Section 4 examines hypotheses and data (or the lack thereof) prior to 1994 for sealing of plasmalemmal holes by direct lipid spread or fusion of plasmalemma leaflets following complete transection of cytoplasmic extensions. Section 5 describes the data for the role of Ca^2+^ and vesicles in plasmalemmal sealing and some unanswered questions such as whether vesicles are moved by bulk flow and/or molecular motors. Section 6 describes various biochemical processes and substances involved in vesicle-mediated natural sealing and emphasize that most are not yet correlated with subcellular events in membrane repair. Section 7 describes and assesses data that do, or do not, support the validity of the plug vs. patch hypotheses and how neither hypothesis for this intermediate event in cell sealing yet accounts for the ultrastructural morphology or biophysical properties of the final state of membrane repair. Section 8 describes how a previous traumatic lesion may facilitate the sealing of a subsequent lesion. Section 9 describes how PEG acts as a direct membrane fusogen and may repair plasmalemmal damage by mechanisms identical to those originally hypothesized prior to 1994. In Section 10, we present our assessment of the current state of the field and suggest experiments that might provide data needed to resolve some current controversies.

## 2 Measures of plasmalemmal sealing and their limitations

### 2.1 Electrophysiological

Membrane potential (V_m_) and input resistance (R_in_) are two measures used to assess the state of a membrane seal in a variety of cell types, although these measures have not been used in most recent studies (see Table 1). These electrophysiological measures typically assume that a restoration of V_m_ and R_in_ to levels of uninjured membranes represent 100% sealing efficacy and lesser values are linearly related to the effectiveness of the seal as a diffusion barrier (Krause et al., 1995; Bittner and Fishman, 2000).

These electrophysiological (V_m_, R_in_) measures of the status of a membrane seal may be reasonably accurate for cells where all plasmalemmal extensions are less than a space constant from the intracellular recording and stimulating electrode(s). However, many cells (or long cytoplasmic extensions such as axons or dendrites) often have membrane cable properties distributed over many space constants. For these cells or cytoplasmic extensions, measurements of V_m_ and R_in_ are often very insensitive to changes in R_in_ at the lesion site, even when measurements are taken within 0.01 of a space constant from the transection or lesion site (Krause et al., 1995). That is, values obtained for R_in_ are often not reliable measures of seal effectiveness (Bittner and Fishman, 2000).

In contrast to measures of R_in_, measures of input current density (I_i_) do not depend upon axon cable properties. Hence, the extracellular measurement of I_i_ by a vibrating probe combined with intracellular measures of V_m_ provide a more reliable measure of the effectiveness of an axonal seal (Krause et al., 1995). Formation of a seal equivalent to that of an intact membrane is considered to have occurred when I_i_ declines to baseline values and V_m_ recovers to values obtained from the uninjured (control) axon. Measures of I_i_ *must* be paired with measures of V_m_ that show that V_m_ is equal to control values for the intact plasmalemma before the cell was injured (Bittner and Fishman, 2000). That is, I_i_ shows a marked decline as V_m_ decreases in an electrically dysfunctional axon with a poorly sealed plasmalemmal lesion. In short, both a recovery of V_m_ combined with a decline of I_i_ to values measured for the intact plasmalemma strongly suggest restoration of barrier permeability to that of the originally intact plasmalemma. Such comprehensive assessments have been made only for completely transected invertebrate giant axons (Krause et al., 1994; Fishman et al., 1995; Eddleman et al., 1997; Eddleman et al., 1998a; Eddleman et al., 2000).

### 2.2 Fluorescent tracers

The extracellular and/or intracellular addition of fluorescent labels have been used to assess the plasmalemma sealing of most cell types reviewed herein. Localization and labeling of dyes are typically observed *via* fluorescence and/or confocal microscopy. The formation of a tracer dye barrier usually correlates well with the formation of a seal as assessed by R_m_, V_m_ and I_i_—and tracer data are easier to obtain than I_i_ (Bittner and Fishman, 2000). However, dye tracers almost always serve as more qualitative than quantitative measures, especially when used to assess the state of a seal in an individually wounded cell (see Section 3.4). When using fluorescent tracers, several variables must be considered to accurately assess the state of a plasmalemmal seal.

#### 2.2.1 Tracer targets: Hydrophilic vs. hydrophobic vs. Ca^2+^

Hydrophilic dye added to the solution bathing an axon is excluded from successfully sealed cells (Eddleman et al., 1997). Alternatively, hydrophilic dye can be loaded into cells by microinjection. If cell-loading is followed by hydrophilic dye addition to the extracellular solution, a clear boundary can be seen between the contrasting dyes (Spötl et al., 1995; Eddleman et al., 1997; Bittner and Fishman, 2000; Chuo et al., 2018). Cells can also be loaded with calcein-AM that is transported into the cell and fluoresces intracellularly after cleavage of the AM moity (Eddleman et al., 1995). Styryl (lipophilic) dye added to the bath prior to wounding is readily incorporated into the plasmalemma and/or myelin (Betz et al., 1992). Labeling the membrane enables observation of endo- and exocytotic events contributing to formation of a seal (Chuo et al., 2018). Fluorescent tracers are also used to monitor ionic dynamics in cells, such as Ca^2+^-sensitive Fura-2 (Steinhardt et al., 1994; Togo, 2019; Sridhar et al., 2020). In oocytes and mammalian cell culture, GFP fusion proteins have been used to study the biochemical agents involved in sealing (Vaughan et al., 2014).

#### 2.2.2 Molecular weight of tracers affect diffusion across plasmalemmal lesions

Barrier formation to exclude the membrane dynamics is associated with sealing (Benink and Bement, 2005; Corrotte et al., 2013). Influx/efflux of various markers depends on the molecular size of the dye molecule used (Gallant and Galbraith, 1997; Eddleman et al., 2000). For example, crayfish MGAs initially exclude Texas Red-dextran (70 kDa, then 3 kDa) followed at least 30 min later by Calcein (0.6 kDa) when the two are added to the extracellular bath (Eddleman et al., 2000; See Section 3.2). Exclusion of ions take yet longer (Krause et al., 1994). Thus, in the cell types in which it has been studied, there is a progressive exclusion to molecules of higher to lower molecular weight as a barrier (seal) forms (reviewed in Vargas and Bittner, 2019).

#### 2.2.3 Electrical charge of tracers affect diffusion across plasmalemmal lesions

Properties of the intracellular environment can affect the uptake of fluorescent tracers. For example, unsealed crayfish MGAs exclude negatively-charged, but not positively-charged, dye molecules (Eddleman et al., 1998b). An outward axoplasmic flow in transected squid GAs can exclude both neutral and negatively charged dye molecules (Eddleman et al., 1998a; Eddleman et al., 2000).

### 2.3 Morphological observation

Morphological observations alone almost always yield ambiguous measures of plasmalemmal sealing, in large part because they usually do not have the resolution to image the 4–10 nm wide membrane bilayer. In general, high magnification (>80,000X) transmission electron microscopy (TEM) is needed to detect whether a membrane is continuous and intact. Even then, it is almost impossible to collect and analyze many serial sections and develop a 3D reconstruction of the membrane topology (Vargas and Bittner, 2019). To the best of our knowledge, this technique has never been utilized to show a continuous membrane as the result of plasmalemmal disruptions more than 1 µm in diameter. Studies claiming *de novo* formation of an intact membrane at the site of injury (See Section 4) using 5000X TEM (Spira et al., 1993) or 100X phase-contrast imaging (Yawo and Kuno, 1985) do not have the resolution to justify a conclusion of a fully formed diffusion barrier.

Scanning electron microscopy (SEM) has also been used to observe the accumulation of vesicles at the transected ends of axons (Eddleman et al., 2002) and lesion sites in sea urchin eggs (McNeil and Baker, 2001) as discussed in detail in Section 4. SEM observations do not, however, provide a reliable measure of the formation of a complete seal due to their inability to monitor in real time paired with measures of dye exclusion or V_m_, R_in_, and I_in_. Additionally, vesicles may collapse or explode as a result of the dehydration and other processes applied during TEM or SEM sample preparation (Chuo et al., 2018; Vargas and Bittner, 2019).

In summary, the most reliable assessments of plasmalemmal sealing use a combination of the measures described above, including serial confocal measures of fluorescent dye exclusion, V_m_ and I_in_, and high-resolution TEM images taken through the lesion site. These multiple-method measures of membrane repair have primarily been studied using invertebrate giant axons (GAs), namely, squid, crayfish, and earthworm GAs (Krause et al., 1994; Ballinger et al., 1997; Eddleman et al., 1997; Godell et al., 1997; Eddleman et al., 1998a; Eddleman et al., 2000; Lichstein et al., 2000; Bittner et al., 2016c; Vargas and Bittner, 2019).

## 3 Use of model systems for studying plasmalemmal repair

### 3.1 Individual echinoderm and amphibian eggs

Unfertilized oocytes of sea urchins (Steinhardt et al., 1994), starfish (Fein and Terasaki, 2005), *Xenopus* (Mandato and Bement, 2001; Clark et al., 2009), and mice (Um et al., 2020), of 50–1000 µm in diameter are often used as model systems to assess sealing of plasmalemmal lesions of less than 1 µm to greater than 5 µm in diameter. Vesicles and other sub-cellular organelles in these cells are easily visualized with fluorescence or confocal microscopy and the cells penetrated with microelectrodes. These large unfertilized oocytes have many intracellular vesicles docked just interior to the plasma membrane and can rapidly interact with each other and the plasmalemma to seal a plasmalemmal lesion within seconds to minutes after natural (sperm) or artificial trauma producing a plasmalemmal hole microns in diameter. Due to such rapid repair, electrophysiological measurements and fluorescent analyses of unfertilized oocytes cannot easily or accurately assess the transition from injury to sealing. Hence, rapid sealing measurements from such unfertilized eggs may lead to inappropriate interpretations of an immediate (step-wise change) barrier formation. The egg models, fertilized or unfertilized, are also often regarded by cell biologists and others as generic representations of eukaryotic cells, compared to axons (described below) that are often regarded as specialized cells not generally representing eukaryotic cells. This assumption may not be appropriate for studies of plasmalemmal repair due to the highly specialized wound response observed in oocytes. That is, almost all other cell types do not have an abundance of docked cortical vesicles and seal within minutes to hours (reviewed in Hendricks and Shi, 2014; Vargas and Bittner, 2019).

### 3.2 Individual invertebrate giant axons

Cytoplasmic extensions (axons, neurites) of invertebrate nerve cells are often used as a model system to assess sealing of small holes or complete transections of membrane-bound structures. These cytoplasmic extensions do not have an abundance of vesicles docked at the membrane (Bittner et al., 2016b). Invertebrate giant axons (GAs) are large enough (50–1,000 µm) to make intra- and extra-cellular electrophysiological recordings from single cells and easily visualize subcellular organelles *via* confocal fluorescence. Additionally, transection of GAs avoids damage to the cell body and its nucleus. Invertebrate GAs have a less rapid sealing response (minutes to hours) to traumatic lesions than unfertilized eggs (seconds to minutes; see Table 1). Hence, the progression of repair processes in invertebrate GAs can be observed with better temporal and spatial resolution. For example, different molecular sized tracers (discussed in Section 2.2.2) are excluded at differing times post transection, a result which would be difficult to impossible measure in rapidly sealing unfertilized eggs (Eddleman et al., 2000). The large size of invertebrate GAs also enables control of some known factors that affect plasmalemmal sealing, such as induction of Ca^2+^-induced vesiculation *via* internal microdialysis of Ca^2+^ (Fishman and Metuzals, 1993). Data from such invertebrate GA models consistently show progressive re-formation of a diffusion barrier similar to an intact plasmalemma, in contrast to the near-instantaneous formation often inferred from oocyte studies (Abe and Cavalli, 2008; Spaeth et al., 2010; Bittner et al., 2016b).

### 3.3 Individual mammalian neurites in culture

Cultured mammalian PC12 or B104 cells as a model system do not exhibit instantaneous sealing and show progressive barrier formation (Yoo et al., 2003), as may most eukaryotic cell types. These cells can be easily individually identified and 20–40 observed in their entirety (i.e., including the nucleus and cytoplasmic extensions) at the same Petri dish. This property can facilitate relatively high throughput comparisons of cell repair under various wounding and environmental conditions. For example, B104 neurites transected closer to the soma successfully seal at lower rates than those transected further away from the cell body (Yoo et al., 2004; Nguyen et al., 2005; McGill et al., 2016). However, these cells are too small in diameter 0.5–2 µm) to easily observe cytoplasmic organelles or penetrate with microelectrodes. These cells are typically utilized to assess data on the biochemical pathways that produce a plasmalemmal seal following neurite transection (Detrait et al., 2000; Spaeth et al., 2012a; Spaeth et al., 2012c; Spaeth et al., 2012b; Zuzek et al., 2012; Poon et al., 2018).

### 3.4 Populations of damaged cells in culture

The use of damaged cells in culture to assess plasmalemmal sealing after traumatic injury involves shearing/scraping of single layer cell cultures (often mammalian epithelial cells) or syringe uptake of sea urchin eggs. In the former procedure, cells are grown to ∼70–90% confluency and the culture dish is scraped to induce widespread, unquantified plasma membrane damage to many cells (Miyake and McNeil, 1995; Terasaki et al., 1997; McNeil and Steinhardt., 2003; Mellgren et al., 2007). In the latter procedure, many sea urchin eggs are taken up into a syringe and subsequently expelled to yield unspecific and unquantified damage to many cells (McNeil and Baker, 2001). The problem in using these models to assess the averaged sealing response is a variety of lesion types across a population of cells of unknown number, likely containing a significant number of cells wounded once or many times and cells without a plasmalemmal disruption.

## 4 Previously-assumed mechanisms of plasmalemmal sealing: Plasmalemmal collapse and lipid spreading

Prior to 1994, the subcellular, biochemical, etc., mechanisms that repair a damaged plasmalemma had not been directly studied. Lipid spreading was often assumed to repair small holes and collapse and fusion of apposed plasmalemmal leaflets were assumed to rapidly seal a complete transection resulting in a continuous plasmalemma whose chemical composition would be similar to the intact membrane (Schlaepfer and Bunge, 1973; Krause and Bittner, 1990; Krause et al., 1994; Vargas and Bittner, 2019).

The sealing of micro-punctures in the plasma membrane were assumed to be repaired by lipid spreading (Kelly, 1985). In this model, the fluid-like mobility of membrane lipids would allow their lateral migration to close the disruption in a thermodynamically favorable process (Figure 1D). However, lateral lipid mobility is now known to be greatly restricted in biological membranes (Golan et al., 1984; Gordon et al., 1985; Kwik et al., 2003) and lipid spreading has only been convincingly demonstrated in artificial unilamellar vesicle membranes (Riske and Dimova, 2005). Nevertheless, lipid spreading was occasionally mentioned as a potential mechanism of plasmalemmal repair (McNeil and Baker, 2001; McNeil and Terasaki, 2001), primarily as a proposal to seal very small plasmalemmal disruptions on nanometer scales (McNeil and Baker, 2001; Horn and Jaiswal, 2019).

Larger-scale plasmalemmal damage of transected cytoplasmic processes was originally assumed to seal by collapse and subsequent fusion of cut plasmalemmal leaflets (Figure 1B) (Kelly, 1985), although no studies were referenced to support the assumption. A few studies on invertebrate axons [squid GAs (Gallant, 1988), cockroach GAs (Meiri et al., 1981), and Aplysia (Spira et al., 1993)] and mammalian axons [rat septal neurons (Xiea and Barrett, 1991) and spinal axons (Kao et al., 1983)] interpreted their data according to this collapse and fusion hypothesis. However, the data in these studies were obtained using techniques that lacked the necessary resolution to support their interpretation (Bittner and Fishman, 2000; Spaeth et al., 2010; Vargas and Bittner, 2019).

One alternative model prior to 1994 arose from studies on the cockroach GA (Yawo and Kuno, 1983; Yawo and Kuno, 1985). This model was based on observations that V_m_ and I_i_ recovered to values representative of a successful membrane seal. Accompanying low-resolution phase contrast microscopy was used to claim the establishment of a high resistance, partition-like bi-layered membrane proximal to the transected end (Figure 1C). This morphometric protocol lacked the appropriate resolution to identify the structure as a lipid bilayer.

## 5 Currently-documented mechanisms of plasmalemma sealing

### 5.1 Ca^2+^-mediated vesicle interactions

The rapid formation of membranous structures has long been reported to occur in response to axonal transection (Ramón Y Cajal et al., 1991). Injury-induced vesicles had been observed to occur after traumatic injury to squid GAs (Fishman et al., 1990). The role of such vesicles was unknown until 1994. In 1994, Krause et al. (1994) reported that transected squid and earthworm GAs seal by an accumulation of vesicles induced by Ca^2+^ influx. That same year, Steinhardt et al. (1994) identified Ca^2+^-induced vesicle accumulation as the primary sealing mechanism in mechanically-wounded unfertilized sea urchin eggs and mammalian fibroblasts. These papers agreed that plasmalemmal repair primarily required influx of Ca^2+^ through a membrane disruption (Krause et al., 1994; Ballinger et al., 1997; Eddleman et al., 1997). Intracellular Ca^2+^ can also increase by influx through voltage-gated ion channels in the plasma membrane (George et al., 1995; Sattler et al., 1996) or release from intracellular stores (See Section 6.3). However, these mechanisms probably secondarily contribute to plasmalemmal sealing/repair, with influx through the lesion site remaining the primary cause of increased intracellular Ca^2+^ (Strautman et al., 1990; Fishman et al., 1995; Ziv and Spira, 1995; Bittner G. D. et al., 2000).

Microdialysis of squid GAs showed that high cytoplasmic concentrations of Ca^2+^ produced vesiculation (Fishman and Metuzals, 1993). Various cellular origins of these Ca^2+^-induced vesicles have been proposed and cells most-likely utilize any membranous structures readily available for plasmalemmal repair (Bittner G. et al., 2000; Bittner and Fishman, 2000). For example, in unmyelinated axons, vesiculation occurs as a result of Ca^2+^ influx from complete transections or mechanically induced lesions that induce membranous structures primarily by endocytosis of the axolemma (Ballinger et al., 1997; Eddleman et al., 1997; Eddleman et al., 1998a). Muscle fibers exhibit formation of dysferlin-containing vesicles derived from the sarcolemma (McDade, Archambeau, and Michele, 2014). Kidney epithelial cells repair plasmalemmal lesions by vesicles formed by a breakup of the SER (Reddy et al., 2001). Transected earthworm GAs often form multilayered vesicles that appear to arise by delaminations of their myelin sheath (Ballinger et al., 1997). Additionally, Ca^2+^ entry causes mitochondria, lysosomes, and preexisting vesicles to migrate towards the injury site (Rintoul and Reynolds, 2010). In more specialized cells, such as oocytes, pre-formed cortical vesicles facilitate repair processes by rapid migration to the lesion site (Terasaki et al., 1997; Mandato and Bement, 2001).

These data summarized above strongly suggest that eukaryotic cells derive vesicles from any and all readily available membranous structures to facilitate wound repair in an evolutionarily conserved mechanism (Eddleman et al., 1997; Spaeth et al., 2010) While the specific final cellular mechanism involved in vesicle-mediated repair (plug vs. patch: see Section 7) is unknown and debated, the biochemical pathways involved (See following section) appear common to sealing of holes and transections in all the model systems reported in Section 4 and mechanisms reported in this Section.

### 5.2 Cytoskeletal reorganization and function

The cortical cytoskeleton has many functions in response to, and repair of, plasmalemmal damage—most prominently aiding in the delivery of vesicles to the lesion site and in the stabilization of the membrane bordering the injury. Plasmalemmal injury disrupts the local cytoskeleton at the wound site and triggers disorder of adjacent structures, perhaps involving active processes of disorganization. For example, microtubule (MT) disassembly is correlated with Ca^2+^ influx at the wound site that directly promotes the detachment of MTs from the plasmalemma (Togo, 2006). Following this disassembly, MTs elongate toward the site of injury. Similarly, Ca^2+^ influx at the wound site drives remodeling of intracellular actin networks by depolymerizing actin filaments to G-actin (Bíró and Venyaminov, 1979; Li et al., 2018; Ebstrup et al., 2021). This reorganization of the cytoskeleton assists the transport kinesin-mediated delivery of vesicles and lysosomes along MTs to the injured portion of plasmalemma (Cabukusta and Neefjes, 2018). MT-independent transport along actin filaments may also occur in long-distance delivery of membranous structures (Schuh, 2011; Ebstrup et al., 2021). For example, actin filaments in muscle cells help deliver membranous structures to sites of sarcolemmal injury (McDade, Archambeau, and Michele, 2014).

In addition to its role in vesicle transport, cytoskeletal reorganization also helps mediate vesicle interactions at the plasmalemma. The cortical actomyosin network facilitates both neuronal and non-neuronal exocytosis (Miklavc and Frick, 2020). Nanoscale remodeling of the cortical actin network aids in the anchoring, priming, and fusion of vesicles with the plasmalemma. Actin networks assist the organization of SNARE proteins and Ca^2+^ channels that comprise the cellular secretory machinery. For example, localization of the synaptic fusion protein SNAP-25 (see Section 6.6) at the plasmalemma depends on the distribution of cortical F-actin in chromaffin cells, (Torregrosa-Hetland et al., 2011).

Furthermore, the cortical cytoskeleton can directly produce mechanical closure of membrane disruptions. In *Xenopus* oocytes, formation of a contractile actomyosin ring (AMR) constricts disruptions by generating tensile forces in the surrounding plasmalemma (Mandato and Bement, 2001). In the AMR, myosin-2 accumulates in a zone bordering the wound edge and F-actin localizes in the surrounding area (Benink and Bement, 2005). The Rho family of GTPases (Cdc42, Rac1, RhoA) act as regulatory proteins in the AMR. Active RhoA colocalizes to myosin-rich regions and phosphorylates the myosin light chain (MLC), increasing actomyosin contractibility. Similarly, Cdc42 associates with F-actin and dephosphorylates the MLC, decreasing contractability. These opposing interactions function to constrict disruptions and decrease the amount of membranous structures required to repair holes in *Xenopus* oocytes (see Darenfed and Mandato, 2011 for review). The authors describe both biochemical processes and sub-cellular events, a rare correlation of biochemical pathways with micro-morphological events in most studies of plasmalemmal repair discussed in Section 6.

Cytoskeletal dynamics in plasma membrane repair are likely a conserved evolutionary process because cytoskeletal components are required for repair in many model systems. For example, F-actin is involved in sealing membrane disruptions in *Drosophila* embryos (Abreu-Blanco et al., 2012) and frog oocytes (Sokac et al., 2003; Ebstrup et al., 2021). However, there are inconsistencies in the precise role of cytoskeletal components. Taxol, an MT stabilizing agent, and cytochalasin E, an actin destabilizing compound, had no significant effect on sealing crayfish MGAs (Godell et al., 1997). In contrast, both substances inhibited sealing of mammalian septal axons (Xiea and Barrett, 1991).

Many aspects of the involvement of the cytoskeleton in membrane repair is not yet well-examined, such as the possible role of cytoskeletal proteins to transport membranous structures to sites of plasmalemmal/axolemmal damage. For example, MTs are disassembled and reassembled towards the membrane disruption (See Section 6.2; Togo, 2006) and may serve to better transport vesicles to the site of injury. Alternatively, vesicles may be carried towards the site of injury by the bulk flow of cytoplasm out of the plasmalemmal disruption.

## 6 Biochemical pathways of plasmalemmal sealing and their biophysical/morphological correlates

In this section, we discuss both well-established and recently identified biochemical pathways and some biophysical/morphological mechanisms that contribute to the formation of a diffusion barrier (seal) at a lesion site.

Numerous parallel biochemical pathways contribute to the formation of a plasmalemmal seal after plasmalemmal lesions. These pathways are initiated by Ca^2+^ influx, along with (perhaps to a lesser extent) release of Ca^2+^ from internal sources (SER, mitochondria), reactive oxygen species (ROS) and mechanical distortion/stress of the plasmalemma (Cai et al., 2009a; Hendricks and Shi, 2014; Cheng et al., 2015). The substances, pathways, and subcellular organelles activated by traumatic injury all converge on one or more cellular biophysical/morphological processes: wound stabilization, cytoskeletal reorganization, and promotion of vesicle-vesicle and/or vesicle-membrane fusion (Figure 3). To date, the intermediate substances in these biochemical pathways are rarely correlated with observations of molecular/cellular events in the sealing process.

### 6.1 Calcium

Intracellular Ca^2+^ concentration ranges from 10^-6^ to 10^-7^ M in most eukaryotic cells (Bagur and Hajnóczky, 2017; Purves et al., 2018). Upon plasmalemmal damage, intracellular Ca^2+^ concentration rises primarily due to influx of extracellular Ca^2+^ at the lesion site along with possible release from intracellular stores. [see Chang and Reynolds (2006) for review]. This injury-dependent rise in intracellular Ca^2+^ initiates most biochemical pathways involved in plasmalemmal sealing, including calpain and other proteases, phospholipase, annexins, and adenylate cyclase (Figure 3). Once activated, these pathways ultimately promote vesicle-membrane interactions and cytoskeletal dynamics (Eddleman et al., 1997; Blanchette et al., 1999; Eddleman et al., 2000; Benink and Bement, 2005). Intracellular Ca^2+^ concentration must be raised (likely above 10^-4^M) to induce sealing in cultured rat hippocampal neurites (Xiea and Barrett, 1991). The plasmalemmal repair process is initiated by this influx of Ca^2+^, rather than plasmalemmal damage *per se* (Bittner et al., 2016b). Therefore, the time course of sealing is properly measured post- Ca^2+^ influx (i.e., addition to media), rather than post-injury (Yoo et al., 2003).

Several substances have been reported to enhance sealing (or sealing processes) in the absence of Ca^2+^. For example, overexpression of TRIM (MG53) in C2C12 cell culture results in the development of filopodia-like structures (Cai et al., 2009a), possibly representing the increased membrane surface area often seen in response to injury. Additionally, PEG (See Section 9) can induce artificial complete and rapid sealing in Ca^2+^-free solutions (Spaeth et al., 2012a; Spaeth et al., 2012b).

### 6.2 Phospholipase pathway

Ca^2+^ influx following injury activates several phospholipases that influence sealing through parallel biochemical pathways (Figure 3). Phospholipase C (PLC) and phospholipase A2 (PLA2) contain Ca^2+^-sensing domains (Leslie and Channon, 1990; Clark et al., 1991), suggesting a role as initiating factors of the phospholipase response. Yawo and Kuno (1983) reported that inhibitors of PLA2 prevented sealing of transected cockroach giant axons. Activated PLA2 yields arachidonic acid (AA) and lysophosphatidic acid (LPA) through cleavage of lysophospholipid and phospholipid, respectively (Hendricks and Shi, 2014).

Novel PKC (nPKC) and conventional PKC (cPKC) are stimulated by AA (Liu and Heckman, 1998; Obis et al., 2015). Injured astrocytes in culture release AA extracellularly for several hours after wounding (Lamb et al., 1997). PKC isoforms (nPKCη and nPKCθ) activate phospholipase D (PLD) (Han et al., 2002; Spaeth et al., 2010). Additionally, both nPKC and cPKC activate myristoylated alanine-rich C-kinase substrate (MARCKS) (Laux et al., 2000). MARCKS can release PIP2 from the plasmalemma, which is cleaved by PLC to yield DAG and IP3 (Goni et al., 2012). IP3 increases cytoplasmic Ca^2+^ by binding to receptors that induce release of intracellular Ca^2+^ stores. PLD is activated by ADP-ribosylation factor (Arf)-1 (Hodgkin et al., 1999), a Rho-family GTPase. PLD releases phosphatidic acid (PA) from phosphatidylcholine (PC) (Hendricks and Shi, 2014). PA then forms DAG through by stimulation of phosphatidylinositol-4-phosphate 5-kinase (PI(4)P 5-Kinase) (increasing PIP2 concentrations) or by direct cleavage through PA hydrolase (Roth, Bi, Ktistakis, and Yu, 1999). LPA released by PLA2 activates Rho signaling. PLC cleaves PIP2, a link between cortical actin and the plasmalemma (Raucher et al., 2000). PIP2 cleavage by PLC may decrease membrane tension by disruption of contractile actin filament arrays, an effect on subcellular structures that has not yet been studied to confirm a correlation of such biochemical and subcellular structural events.

### 6.3 Oxidation pathway

Upon plasmalemmal disruption, reactive oxygen species (ROS) are produced by, or are affected by, cytoplasmic reactions and organelles (Spaeth et al., 2012). For example, the mitochondrial Ca^2+^ uniporter takes up excess Ca^2+^ following wounding. Increased Ca^2+^ produces a transient increase in the mitochondrial production of ROS (Cai et al., 2009a; Cai et al., 2009b). An increase in ROS activates the GTPase RhoA, triggering the accumulation of F-actin at the injury site (Figure 3). Inhibition of the mitochondrial Ca^2+^ uniporter or ROS significantly decreases plasmalemmal sealing in mouse C2C12 myoblasts (Horn et al., 2017). RhoA recruits and activates both mDia1 and Rho-associated protein kinase (ROCK). The activated mDia1 promotes actin polymerization while ROCK phosphorylates the MLC, increasing actomyosin contractibility (Boyer et al., 2006; Sarasa-Renedo et al., 2006). The interactions of mDia1 and ROCK are identical to those in the formation of a contractile AMR (Section 6.2), suggesting redundancy in the regulatory signaling involved in plasmalemmal repair.

ROS also regulate pathways that yield vesicle-membrane interactions similar to those activated by Ca^2+^. In cultured muscle cells, activation of Tri-Partite Motif (TRIM) family proteins is oxidation-dependent (Cai et al., 2009a). TRIM72 (also known as MG53, a muscle-specific protein) accumulates at phosphatidylserine (PS)-rich regions of the plasmalemma and on the surfaces of intracellular vesicles, alongside Dysferlin and Caveolin-3 (Cav3) (Cai et al., 2009a). Activation of TRIM family proteins by ROS enhances sealing through interaction with synaptic fusion proteins (Spaeth et al., 2012c). Additionally, ROS induce surface-bound MG53 oligomerization (Boucher and Mandato, 2015) and thus may enhance the tethering of vesicles to each other and to the plasmalemma during plug formation (i.e., facilitating vesicle-vesicle interactions). Specifically, mini-dysferlin72 associates with MG53 at the plasmalemma disruption, suggesting involvement of calpain-mediated cleavage of full length dysferlin (Lek et al., 2013). Crosslinking of vesicles and/or the membrane *via* MG53 oligomerization serves as a prime example of a biochemical interaction being related directly to cytoplasmic biophysical/morphological mechanisms of plasmalemmal repair.

### 6.4 Calpains

Calpains (Figure 3) are a family of Ca^2+^-dependent proteases that cleave cytoskeletal substances that then release membrane bound PLC and PKC. Calpain-activated PLC increases diacylglycerol (DAG) and IP3 through cleavage of PIP2 (Zuzek et al., 2012). PKC increases intracellular [DAG] by phosphorylating MARCKS. Activation of endogenous calpains by elevated cytoplasmic Ca^2+^ concentration enhances proteolysis of cortical cytoskeleton proteins (Siman, Noszek, and Kegerise, 1989). Targets of Ca^2+^-activated calpain include dysferlin, AHNAK, and spectrin (Huang et al., 2008). Axons express a network of F-actin filaments interconnected by an association with spectrin parallel to the plasmalemma (Xu, Zhong, and Zhuang, 2013) which could act as a barrier to endocytic events. Hence, cleavage of cytoskeletal linking proteins by calpains may enhance plasmalemmal sealing by decreasing membrane tension, similar to the action of PLC (Section 6.2).

Similar to their role in regulating actin dynamics, calpains target MT-associated proteins (Johnson, Litersky, and Jope, 1991). Talin, which interacts with actin capping proteins to promote cytoskeletal reorganization, is cleaved by calpain (Camussi et al., 2010). Calpain may be necessary for sealing since transected squid giant axons do not completely seal when monitored up to 60–120 min post-transection without the addition of exogenous calpain (Godell et al., 1997). Similarly, the sealing of transected crayfish MGAs is impaired by inhibitors of exogenous calpain. Deletion of calpain-encoding genes in mouse embryos decreases their ability to seal (Mellgren et al., 2007).

### 6.5 Annexins

Annexins are a family of proteins (Figure 3) that exhibit Ca^2+^-dependent binding to negatively charged membrane phospholipids (PS, PA, and PI) (reviewed by Gerke et al., 2005; Boye and Nylandsted, 2016). Annexins show a graded response to cellular wounding and Ca^2+^ influx, with AnxA6 and AnxA2 being the most sensitive to Ca^2+^ and earliest acting in the sealing process. AnxA1 and AnxA5 are activated later when intracellular Ca^2+^ concentration is increased (Rosengarth et al., 2001; Monastyrskaya et al., 2007). Following Ca^2+^-influx, AnxA1, and AnxA2 hetero-tetramerize with S100 proteins. The AnxA2/S100A10 complex associates into a multimeric complex with AHNAK in a Ca^2+^-independent manner (Benaud et al., 2004) and binds dysferlin localized at the injury site (Huang et al., 2007). AnxA2 associates with the actin bundles that tether secretory granules to the plasmalemma in chromaffin cells (Gabel and Chasserot-Golaz, 2016). This complex may enhance plasma membrane stabilization and/or actin cytoskeletal remodeling (Demonbreun et al., 2016).

Similarly, AnxA5 at membrane edges surrounding the wound site associates with PS to form a stable, two-dimensional array that may prevent wound expansion in oocytes (Bouter et al., 2011). AnxA5 is required for sealing of human myotubes (Carmeille et al., 2016). The role of AnxA5 in wound stabilization is an important example of a correlated biochemical and cellular events lacking for most substances in various pathways.

Membrane blebbing has been proposed as an alternate mechanism to seal small (<100 nm) plasmalemmal holes (see Koerdt, Ashraf, and Gerke, 2019 for review). Membrane blebs incorporate injured membrane into a vesicle-like structure that is sealed off from the rest of the cytoplasm by an AnxA1 plug (Draeger, Monastyrskaya, and Babiychuk, 2011). The cellular utilization of this mechanism other than for non-traumatic, toxin-induced, pore expression is yet unknown.

### 6.6 cAMP pathway

Increased intracellular Ca^2+^ concentration upregulates cyclic AMP (cAMP) that activates both PKA and Epac1/Epac2 (Spaeth et al., 2012a; Spaeth et al., 2012b; Spaeth et al., 2012c; Zuzek et al., 2012; Togo, 2017). Activated PKA increases the activity of downstream synaptic fusion proteins such as synaptotagmin VII and SNAP-25 (Yoshihara, Suzuki, and Kidokoro, 2000). In a parallel pathway (Figure 3), the guanine exchange factors (Epac1 and 2) facilitate activation of both synaptic fusion proteins and Golgi fusion proteins. Epac produces an increase in neurotransmitter release in rat hippocampal (Ster et al., 2009), PC12 (Hatakeyama et al., 2006), and B104 cells (Spaeth et al., 2012c). Epac also activates Rap1, an upstream effector of Cdc42 (de Rooij et al., 1998). Both Rap1 and Cdc42 accumulate at PIP2 and PIP3 rich portions of the plasmalemma (Johnson, Erickson, and Cerione, 2012). This accumulation ultimately serves to localize dynamic branched F-actin adjacent to the disruption through activation of neutral Wiskott-Aldrich syndrome protein (N-WASP) and then Arp2/3 proteins (Rohatgi et al., 1999). This evolutionarily-conserved mechanism of branched actin formation regulates membrane tension and curvature, as well as facilitates clathrin-mediated endocytosis (for review see Papalazarou and Machesky, 2021). Branched action filaments are mechanosensitive, perhaps aiding in the initiation of repair processes in response to stretch injuries.

### 6.7 DAG

Activation of the phospholipase and calpain pathways increases the localized concentration of DAG in raft sections of a plasmalemma. DAG activates proteins involved in neurotransmitter release (Zuzek et al., 2012), perhaps by reducing hydration of bilayer surface substances (Goni and Alonso, 1999) and facilitating vesicle fusion and/or vesicle budding (Hendricks and Shi, 2014). DAG activates cPKC and nPKC by interacting with their C1 domain (Liu and Heckman, 1998; Goni et al., 2012). DAG can be metabolically altered to produce several membrane phospholipids, including PS and PC, that affect membrane stabilization and curvature (Millan-Oropeza, 2017). Finally, DAG produces vesicle exocytosis *via* its interactions with Munc13-1 and Doc2α, two synaptic fusion proteins (Mochida et al., 1998). Much of DAG’s role in membrane repair involves altering membrane-specific properties, such as membrane curvature. However, *in vivo* evidence is lacking for any advantage conferred by DAG in the membrane repair processes, such as enhanced vesicle formation or specific downstream interactions.

### 6.8 ESCRT

Endosomal Sorting Complex Required for Transport (ESCRT) accumulates at sites of nanometer-sized plasmalemmal holes until sealing is complete (Jimenez et al., 2014) and is one of the few examples where a substance in the sealing pathway has its biochemical pathway correlated with specific biophysical/morphological cellular effects (Table 2; Figure 2). The ESCRT machinery is assembled at the site of injury, initiated by the Apoptosis-Linked Gene 2 (ALG-2)—a Ca^2+^-binding protein. ALG-2 associates with specific ESCRT subunits beginning with ALG-2-Interacting protein, followed by ESCRT-III and Vps4 (Scheffer et al., 2014). AnxA7 may help recruit ALG-2 to a site of plasmalemmal damage (Cai et al., 2009a). Filaments composed primarily of ESCRT-III form at the plasmalemma and promote topographic changes in the membrane followed by scission (reviewed by Schoneberg et al., 2016). ESCRT complexes seal small (<100 nm wide) plasmalemmal holes generated by mechanical or laser-induced micro-punctures (Jimenez et al., 2014), or by pore-forming toxins, which produce stable protein multimers that span the plasma membrane. Given the small diameters of these plasmalemmal wounds and the role of ESCRT proteins and adaptors in endosomal processes, ESCRT proteins may have auxiliary roles to help seal complete transections or larger plasmalemmal holes caused by traumatic injury (Moe et al., 2015).

**TABLE 2:** Results of sealing experiments.

	Invertebrate GAs	Oocytes	Mammalian neurites	Mammalian epithelial cells	Muscle cells
Sealing time	Squid GA: ∼50min^a^ ^(4,9,10,12)^	*Xenopus* oocyte: <30–60s^(14,18)^	Rat astrocytes: ∼15min^(19)^	HeLa: ∼300–400s^(44H)^	C2C12 Myoblasts: 100–200s^(55,56)^
	Earthworm GA: ∼60min^(1,10,11)^	Unfertilized sea urchin egg: 15–30s^(15,16,17)^	B104 cells: 2–6min^(26,28,29,30)^	MDCK: ∼50s^(52,53)^	Mouse myofibers: ∼160s^(59)^
	Crayfish MGA: ∼60min^(4,6,9)^		PC12 cells: 5–10 min^(29)^	3T3 Fibroblasts: ∼90s^(51)^	
Ca^2+^ requirement	1,2,3,4,5,7,9,10,12	14,15,16,17,18	20,23,24,25,26,28,29,30	35,37,43^H^,47,50^H^,52,53,54	55,58,59
Annexins	–––––––––	14	20,22	31,32,35,38,39,4^H^	57^H^,59,62,66^H^
Oxidation pathway	9	14	19,24,25,27	42,48,49	55,56,57^H^,60,61^H^,62,63^H^,64^H^,66^H^
Phospholipase pathway	3,9	14,18	24,30	46,51	–––––––––
Calpains	9	–––––––––	29	42,47	61^H^,63^H^
cAMP	3,9	14,18	19,21,24,27,30	33,46,51,52	–––––––––
ESCRT	–––––––––	–––––––––	–––––––––	34^H^,44	65
Synaptic fusion proteins	3	16	22,23,26,27,29,30	–––––––––	64^H^
Changes in local lipid composition	–––––––––	13,14,18	30	45,48,50^H^	55,59
Cytoskeletal dynamics	9	16,18	24	31,33,36,37,41^H^,43^H^,47,54	55,58,60

**Sealing times and biochemical pathways found to be involved in the sealing process across model systems.** Numbers in the table represent original research articles publishing these results (see Supplemental Materials one for the full list). A superscript H (X^H^) indicates that the referenced paper used human cells as a model system.

a Complete seal formation has only been observed in squid GAs treated with exogenous calpain

### 6.9 NSF

Pathways facilitating activation of both Golgi and synaptic fusion proteins ultimately converge onto N-ethylmaleimide (NEM)-sensitive factor. Activation of oxidation, PKA, and Epac pathways simultaneously cannot overcome inhibition of NSF by NEM treatment (Spaeth et al., 2012a; Spaeth et al., 2012b; Spaeth et al., 2012c; Zuzek et al., 2012), confirming that NSF acts downstream of these sealing pathways in B-104 cells. NEM also inhibits NSF-induced fusion of membranes in rat neurites (Garcia et al., 1995).

### 6.10 Lysosomal and enlargeosomal exocytosis

Lysosomal exocytosis may function as an initial response to wounding by expanding the pool of available vesicles for plasmalemmal sealing. Lysosomes fuse with the plasma membrane following an influx of Ca^2+^. Acid sphingomyelinase generates ceramide by cleavage of sphingomyelin, which promotes negative membrane curvature (Holopainen, Angelova, and Kinnunen, 2000) and thus inward budding of the plasmalemma (Tam et al., 2010; Corrotte et al., 2013). Cav1 or Cav3 are required for this endocytic internalization (Corrotte et al., 2013). Both Cav3 and dysferlin are downstream targets of TRIM family proteins/MG53 (Section 6.3). In adult muscle cells, dysferlin is rapidly recruited to the site of injury in an actin-cytoskeleton-dependent manner and is required for endocytosis (McDade, Archambeau, and Michele, 2014). Exocytosis of lysosomes involves synaptotagmin VII (Martinez et al., 2000), a synaptic fusion protein which binds syntaxin at intracellular Ca^2+^ levels considerably lower than other synaptogamin isoforms (Krasnov and Enikolopov, 2000). Given the low Ca^2+^-concentration dependency of many fusion proteins, this response is likely initiated rapidly and functions early in the sealing timecourse.

Another rapid initial response to wounding may involve the exocytosis of small (<100 nm diameter) vesicles (enlargeosomes). First identified in PC12 clones (Borgonovo et al., 2002), enlargeosomes fuse to the plasmalemma, thereby increasing the surface area and in turn decreasing the membrane surface tension. Enlargeosomal fusion may be mediated by synaptic SNARE substances including VAMP4 (Cocucci et al., 2008). AnxA2 has also been implicated in this specific exocytotic process (Chasserot-Golaz et al., 2005). Enlargeosomes would function to increase the amount of membrane area available for repair vesicles and to decrease membrane tension.

### 6.11 Removal of pore-forming toxins

In addition to mechanically-induced membrane wounds, the plasmalemma can be disrupted by the insertion of stabilized membrane pores. Bacterial toxins such as streptolysin O damage cells through the insertion of large transmembrane pores that induce cell death and/or nutrient release (reviewed in Boucher et al., 2019). These pore-forming toxins (PFTs) allow for uncontrolled influx of Ca^2+^ into the target cell.

Eukaryotic cells do not repair these injury types by the accumulation of vesicles, but rather by the excision of surrounding membrane (Iacovache et al., 2012). PFTs are removed by either shedding of membrane blebs or endocytic degradation. Ca^2+^ influx through PFTs activates biochemical pathways that are shared with vesicle-mediated membrane repair. Formation of membrane blebs is promoted by calpain-mediated disruption of the cortical cytoskeleton (Atanassoff et al., 2014) and/or selective insertion of PFTs into cholesterol-rich regions of the plasmalemma resulting in changes to membrane curvature (Drucker et al., 2018). PFT removal involves the recruitment of annexins to PFT sites that produce cytoskeletal changes that contract the neck of forming membrane blebs (Babiychuk et al., 2009; Atanassoff et al., 2014).

Lysosomal endocytosis (see Section 6.10) may also be involved in the removal of PFTs from the plasmalemma. PFT permeabilization results in increased sphingomyelinase activity, favoring negative membrane curvature through the generation of ceramide in the plasmalemma (Walev et al., 2001). The endocytotic mechanism of PFT removal of inward membrane curvatures is reported to occur *via* active Ca^2+^-dependent processes and/or *via* passive Ca^2+^-independent processes (Boucher et al., 2019). PKC and p38 MAPK is upregulated upon membrane disruption by PFTs (Stassen et al., 2003), perhaps further directing changes in local membrane composition through the downstream effect on the DAG pathway (see Section 6.7).

## 7 Current models of plasmalemmal sealing

The pathways and cellular events described above and in Figures 2, 3 are consistent with vesicle-mediated, biophysical/morphological sealing mechanisms in which the influx of Ca^2+^ through the lesion site causes the formation of membranous structures that form a diffusion barrier at the lesion site. Two biophysical/morphological mechanisms (patch and plug) have been proposed to explain the initial seal (see Figure 2). These two hypothetical mechanisms involve the same initial vesicular interactions and biochemical pathways, but differ in the final events that form a complete functional barrier that has the diffusion characteristics of the original intact plasmalemma (Bittner and Fishman, 2000; Davenport et al., 2016) but not its morphological structure (Lichstein et al., 2000). In fact, the two biophysical/morphological sub-cellular mechanisms may be distinctions without a difference in terms of outcome, given that both ultimately form a diffusion barrier that approximates the permeability and ultrastructure of an undamaged plasma membrane. In the following subsections, we describe each model and examine the evidence for and against both hypothesized biophysical/morphological mechanisms.

### 7.1 The vesicle patch model

As proposed for *Xenopus* oocytes and sea urchin eggs, the vesicle patch model hypothesizes a one-step membrane sealing mechanism in which vesicles accumulating at the wound site first fuse together to form a large membranous “patch” which then completely repairs the plasmalemmal hole or transection in a single fusion step. This theoretical biophysical/morphological mechanism is based primarily on an observation that Ca^2+^-containing sea water injected into a sea urchin egg is quickly contained within a newly formed “wound vesicle” (Terasaki et al., 1997). A good schematic figure for this patch mechanism (Davenport et al., 2016) is derived from indirect data that are somewhat controversial (Bittner and Fishman, 2000; Bittner et al., 2016b; Vargas and Bittner, 2019). For instance, McNeil and Baker (2001) show several images of limited vesicle-vesicle fusion events—but do not publish an example of a complete wound patch. Davenport et al. (2016) proposes the process of “explodosis” in which the outer membrane layer of the patch opens to yield a single bilayer, shown only in a schematic figure. This cellular event has not been directly observed and published.

If a plasmalemmal disruption seals in a single step, one would expect to see a stepwise change in V_m_, R_in_, I_i_, and dye exclusion. However, all reports of these functional measures indicate a progressive and gradual change in other sealing models that do not have an abundance of pre-formed, sub-plasmalemmal vesicles (Bittner and Fishman, 2000; Vargas and Biitner, 2019). Furthermore, the rate and extent of dye exclusion depends inversely on the dye molecular size in transected crayfish MGAs, consistent with a progressive formation of a seal (Eddleman et al., 2000).

### 7.2 The vesicle plug model

In contrast to a single-step patch seal by a wound vesicle, the vesicle plug hypothesis assumes that plasmalemmal holes or complete transections of cytoplasmic extensions are sealed by an accumulation of vesicles which more gradually forms a diffusion barrier through vesicle-vesicle and vesicle-plasmalemmal interactions. Vesicles and other membrane bound structures (single and multilayered vesicles, lysosomes, mitochondria, etc.) interact with each other and the plasmalemma at the lesion site to form a “vesicular plug,” that form a diffusion barrier to particles of smaller and smaller molecular size. Several biophysical/morphological mechanisms likely play a role in the formation of a vesicle plug, including:

a) Dense packing of vesicles at the site of injury creates a longer and narrower diffusion path, possibly involving vesicle to vesicle attachment proteins such as gap junctions, adherens, and desmosomes (Bittner and Fishman, 2000; Fishman and Bittner, 2003):
b) Contraction of an actomyosin ring (See Section 6.2) surrounding the plasmalemmal disruption may account for the observed narrowing of transected GAs (Krause et al., 1994; Bittner and Fishman, 2000; Eddleman et al., 2002). Additionally, contraction is suggested to help seal small plasma membrane holes (Mandato and Bement, 2001; Benink and Bement, 2005). However, membrane contracture has not been demonstrated to yield complete membrane closure/sealing.
c) Internalization or budding of membrane, mediated by ESCRT. Endocytosis could remove and subsequently seal small (<100 nm) plasmalemmal disruptions (Jimenez et al., 2014; Moe et al., 2015).
d) Fusion of vesicles to form larger or even multi-layered vesicles that would continue to accumulate at the wound site. In crayfish MGAs, squid GAs, and earthworm GAs, plasmalemmal injury induces formation of single-walled vesicles of plasmalemmal origin and multi-walled vesicles likely of glial origin (Ballinger et al., 1997; Eddleman et al., 1998a).

Via a combination of these events and vesicle accumulation, a vesicular plug is hypothesized to progressively form a diffusion barrier at the plasmalemmal lesion (Krause et al., 1994; Eddleman et al., 1997; Eddleman et al., 1998a; Eddleman et al., 2000; Fishman and Bittner, 2003) The following results are consistent with the vesicle-plug hypothesis.

a. Morphological observations show a gradual accumulation of membranous structures at sites of disruption associated with a diffusion barrier to smaller and smaller molecules (Fishman and Bittner, 2003) rather than a one-step sealing event. SEM observations of squid GAs and crayfish MGAs show a non-uniform accumulation of vesicles at the cut end of axons (Eddleman et al., 2002). Furthermore, SEM of unfertilized sea urchin eggs reveals heterogeneous massing of membranous structures following injury (McNeil and Baker, 2001).
b. Sealing is slower in MGAs and other cells that do not have large populations of pre-formed vesicles directly below the plasmalemma such as *Xenopus* oocytes and unfertilized sea urchin eggs (Bittner G. D. et al., 2000; Fishman and Bittner, 2003). These numerous vesicles in unfertilized eggs rapidly migrate to the injury site and form a diffusion barrier within seconds (Steinhardt et al., 1994; Terasaki et al., 1997).
c. The sealing of smaller lesions is more rapid than larger lesions. PC12 cells (Yoo et al., 2004) and the neurites of B104 cells (McGill et al., 2016) show much slower sealing times than that of crayfish MGAs and squid GAs (Bittner and Fishman, 2000; Fishman and Bittner, 2003).
d. The time to exclude tracer molecules varies inversely with molecular weight and molecular diameter of the tracer. Larger fluorescent dye molecules placed extracellularly are initially excluded followed by the exclusion of tracers of smaller and smaller size (Eddleman et al., 2000). Furthermore, ions of yet-smaller molecular weight and size are excluded later than small fluorescent dyes of higher molecular weight (Eddleman et al., 1998a).
e. Probability of sealing varies monotonically and continuously with the time at which Ca^2+^ influx is initiated. When cells are transected in Ca^2+^-free physiological saline and then placed in a Ca^2+^_-_containing solution, dye barriers form at similar post-calcium addition times rather than at similar post-transection times (Yoo et al., 2004). Furthermore, sealing time increases with distance of the lesion site from the soma (Yoo et al., 2004; Nguyen et al., 2005).

Together, these results provide evidence against a one-step sealing event that should be produced by a wound vesicle. In contrast, these results are consistent with the vesicle plug hypothesis, in which vesicle accumulation followed by vesicle-vesicle and/or vesicle-membrane interactions progressively form a seal.

Future research to resolve this controversy could use more powerful current microscopy and labeling techniques. Recently-enhanced time-lapse confocal microscopy and three-dimensional reconstruction of images should reveal mechanisms of vesicle accumulation and interaction in various cell types (including invertebrate GAs and unfertilized oocytes). Modified SEM techniques that eliminate artifacts due to oxidation during fixation (Chuo et al., 2018) could distinguish between patch vs plug mechanisms of vesicle accumulation. The combined use of current confocal and SEM imaging could also determine if the mechanism of seal formation is universal among all eukaryotic cells or is cell-specific, i.e., determined by cell morphology and the immediate availability of membranous structures to seal a damaged plasmalemma.

### 7.3 Reestablishment of a continuous lipid bilayer

Formation of a vesicle seal—either through the patch or plug hypothesis—results in a membrane topology very different from that of its uninjured state. SEM images of both crayfish MGAs (Eddleman et al., 2002) and unfertilized *Xenopus* oocytes (McNeil and Baker, 2001) show collections of variously-sized membranous structures (0.5–6 µm on average) jutting out from the injury site. Furthermore, successful vesicle-mediated repair changes the spatial distribution of lipid species and proteins in the plasmalemma. Following injury, microdomains (rafts) enriched with specific lipid species such as DAG and sphingomyelin form to facilitate repair processes (see Section 6.7 and Section 6.10; Holopainen, Angelova, and Kinnunen, 2000; Hendricks and Shi, 2014). It is likely that the distribution of membrane-associated proteins is similarly disrupted following injury. Thus, cell wounding and subsequent vesicle-mediated repair results in a diffusion barrier with a vastly different topology and distribution of proteins and other substances compared to its native (uninjured) state.

The native distribution of lipid species throughout the plasmalemma must presumably be eventually re-established following initial seal formation (Sønder et al., 2021). While a continuous membrane bilayer resembling that of the uninjured cell is observed at 24 h following injury (Lichstein et al., 2000), no long-term imaging studies of injured cells have been performed to this date. Reestablishment of the native membrane lipid composition is also currently unknown and might occur by (1) lateral flow of vesicle-lipids into the seal surround as vesicles begin to collapse into the plasmalemma or (2) the *de novo* synthesis and integration of membrane lipids into the plasmalemma in conjunction with removal and breakdown of repair vesicles. New techniques in *de novo* lipid labeling combined with time-lapse confocal microscopy could be used to determine the contribution of lipid metabolism to restoration of a membrane bilayer.

## 8 Adaptive responses following a conditioning plasmalemmal sealing event

Uncontrolled Ca^2+^ influx at sites of membrane disruption produces rapid degeneration of cellular structures and eventually cell death (Schlaepfer and Bunge, 1973; Trump and Berezesky, 1995). The ability of a cell to retard this Ca^2+^ influx and re-form a membranous barrier to the extracellular environment is crucial to its survival. As described in previous sections, eukaryotic cells repair plasmalemmal lesions and prevent apoptosis by evolutionarily conserved subcellular mechanisms using vesicular interactions and biochemical pathways. This section describes adaptive responses following plasmalemmal injuries that are common to many eukaryotic cell types that enable them to prevent further injury and/or more rapidly seal a second plasmalemmal injury.

**FIGURE 4:**
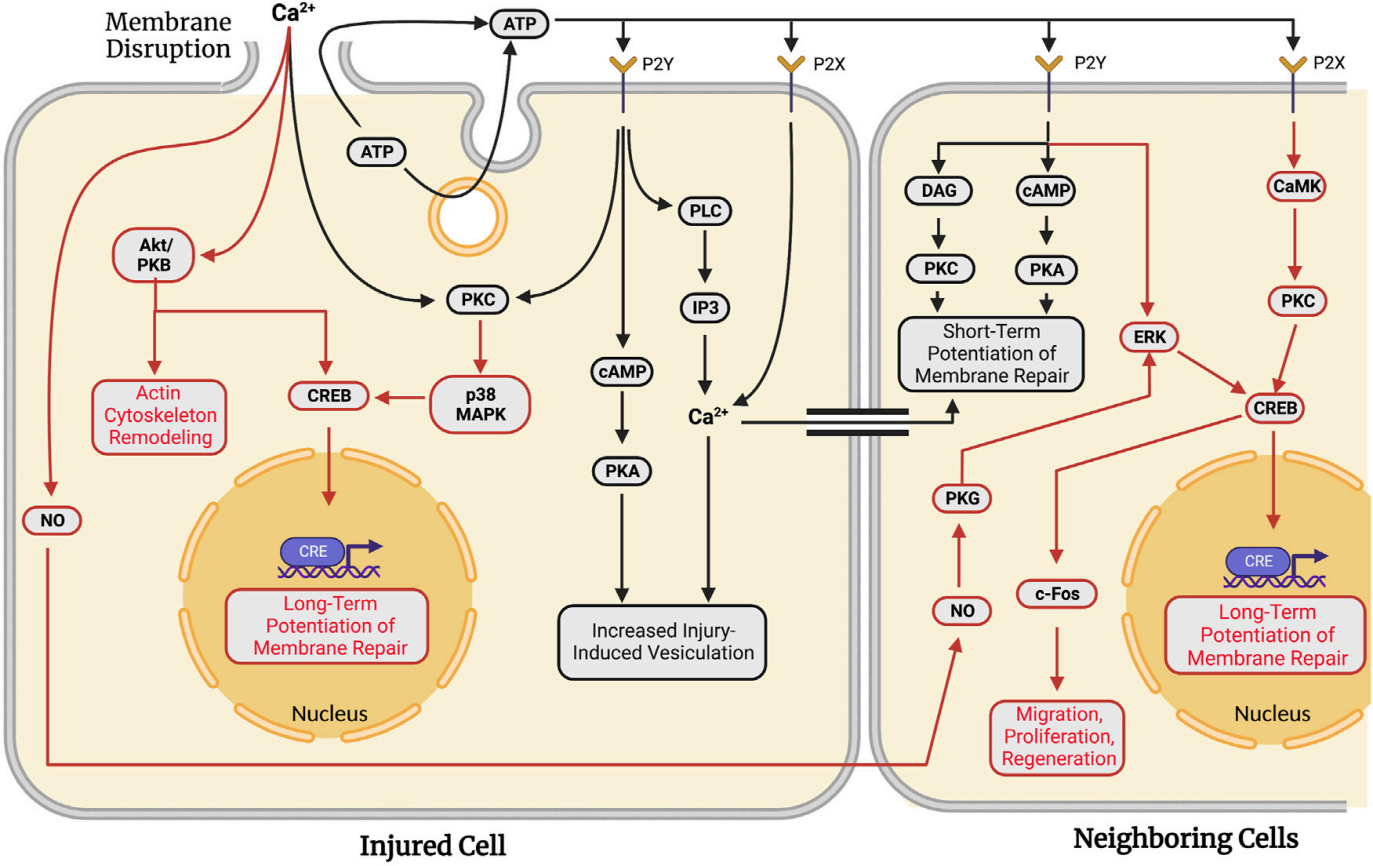
Adaptations against future wounding events in injured and neighboring cells in response to traumatic membrane injuries. Successful repair of a plasmalemmal disruption in a given single cell causes both short-term (facilitated response, black lines) and long-term (potentiated response, red lines) adaptations to increase the probability of survival following subsequent injury to that cell. In addition to these “adaptive” responses for the wounded cell, signaling pathways are activated to produce similar adaptations in multi-cellular systems in which surrounding cells are also injured. Neighboring cells exhibit short- (black lines) and long-term (red lines) potentiation of membrane repair processes. In both single cells and populations of injured cells, long-term potentiation involves transcription factor-mediated upregulation of genes associated with plasmalemmal repair and cell survival. Note that many biochemical pathways and substances are common to plasmalemmal repair of traumatic lesions to single cells (see Section 6) and adaptive response pathways in the same cell or neighboring cells.

After wounding, cells display short-term and long-term adaptations to respond more rapidly to future wounding in the damaged cell and in neighboring undamaged cells (reviewed in Togo, 2019). Short-term responses can involve an increase in endocytosis, expansion of vesicle pools, and migration of available membranous structures to plasmalemmal lesion sites. Long-term responses involve the activation of transcription factors that regulate cell survival. Figure 4 summarizes the primary biochemical pathways involved in the diverse set of adaptive responses activated following the successful formation of a membrane seal to increase cell survival in the event of a subsequent membrane injury.

MDCK cells wounded for a second time in tissue culture at the same site of a previous injury exhibit resealing rates nearly double that of cells wounded a single time (Togo, 2012), i.e., a facilitated short-term adaptive response. This short-term adaptive response activates PKC, possibly leading to phosphorylation of proteins involved in exocytosis, similar to those depicted in Figure 3 (Togo et al., 1999; Togo et al., 2003). This short-term facilitation is sensitive to Brefeldin A, suggesting the involvement of Golgi fusion proteins. The facilitated response also exhibits the reorganization of microtubules (Togo, 2006; see Section 6.2 for further details).

This potentiated short-term resealing response occurs when a cell is wounded at a second site at least 10–20 µm from the initial injury site. This short-term potentiated response requires PKA, but not PKC (Togo et al., 1999; Togo et al., 2003; Togo and Steinhardt, 2004). ATP is released extracellularly either by exocytosis or diffusion through the membrane disruption (Togo, 2017). Extracellular ATP activates P2Y G-protein-coupled receptors and P2X ligand-gated ion channels (Burnstock, 2007). P2Y receptors cause a release of Ca^2+^ from intracellular stores (*via* PLC and IP3), increased PKC activity, and increased cytosolic cAMP concentrations (Balboa et al., 1994; Post et al., 1998). In contrast, activated P2X receptors produce a direct influx of Ca^2+^. The primary effect of these receptors is to activate the cAMP pathway. cAMP activates PKA and a subsequent increase in injury-induced vesiculation (Togo, 2019).

These short-term responses in wounded MDCK cells are also seen in neighboring cells within 5 min of the initial injury event (Figure 4). When injured, neighboring cells seal faster than the initially injured cell (Togo, 2014). This potentiation is observed up to 6 cells distant to the initially wounded cell (Togo, 2012) and is primarily attributed to the extracellular release of ATP from the initially wounded cell. Inhibition of extracellular ATP signaling eliminates the potentiated response in neighboring cells (Togo, 2017). ATP activates both a DAG and cAMP pathway in neighboring cells *via* binding to P2Y receptors, similar to pathways observed in non-adaptive repair (Section 6.6 and Section 6.7). These pathways activate PKC and PKA, yielding a short-term potentiated repair response in locally neighboring cells. Diffusion of calcium through gap junctions between wounded and neighboring cells may also induce potentiation.

Loading cells with styryl dye reveals an important difference between facilitated and potentiated short-term responses. Upon initial membrane disruption, localized vesiculation is primarily observed immediately surrounding the disruption. Wounding twice at the same site produces site-specific (local) vesiculation while subsequent wounding at a different site yields global vesiculation throughout the cell (Togo et al., 2003). The initial wounding of a cell activates several signaling pathways and increases the cell’s capacity to seal additional membrane disruptions by the rapid formation of membranous structures.

In addition to the short-term adaptive resealing responses in wounded cells, long-term adaptations occur in both wounded and neighboring cultured MDCK cells. Both long- and short-term responses involve the phosphorylation of CRE-binding protein (CREB) that alters transcription. In 3T3 fibroblasts, membrane disruption stimulates PKC cascades. PKC then activates a p38 MAPK pathway that ultimately leads to CRE-mediated gene expression (Togo, 2004). PKC and p38 MAPK are also upregulated upon membrane disruption by pore-forming toxins (Stassen et al., 2003). Long-term responses may involve Akt/PKB which has numerous downstream effectors regulating cell survival (see Song, Ouyang, and Bao, 2005 for review). Akt/PKB activates CREB to stimulate transcriptional control of apoptosis-related genes. Akt/PKB is localized to the cytoskeleton upon membrane disruption to interact with actin and Cdc42 (Cenni et al., 2003; see Section 6.2). Consistent with this result, injured cells often activate c-fos, a target of phosphorylated CREB (Sassone-Corsi et al., 1988; Grembowicz et al., 1999).

Long-term adaptive responses are also observed in neighboring cells. Diffusible signaling molecules such as nitric oxide (NO) are released from wounded cells (Togo, 2012). NO/PKG signaling stimulates potentiation of membrane repair in neighboring cells. PKG stimulates CREB phosphorylation through ERK (Ciani et al., 2002). Similarly, extracellular ATP can stimulate ERK through activation of P2Y receptors (Neary et al., 2005). ATP-dependent activation of Y2X receptors can phosphorylate CREB through a PKC and CaMK pathway (Tan et al., 1996). ESCRT machinery has been proposed to generate extracellular vesicles that play a role in intercellular signaling following membrane disruption (Scheffer et al., 2014). Once activated, CRE in neighboring cells functions in a very similar manner to that described in a singly-wounded cell, i.e., primarily to regulate long-term potentiation of membrane repair. Other adaptive capabilities may include regulation of cell migration, survival, and regeneration (see Togo, 2019).

Although relatively few adaptive membrane repair responses have been studied, the adaptive pathways and substances involved are similar to those reported for the repair of single plasmalemmal lesions. The biochemical pathways reported have not yet been correlated with observations of sub-cellular biophysical/morphological events, as is also the case for many single cell traumatic studies reported in previous sections.

## 9 Artificially-induced plasmalemmal repair

In addition to the natural methods of plasmalemmal sealing described in Sections 5–8, cell membranes can be fused or sealed by PEG, a 1–5 kDa synthetic amphoteric polymer that is commonly used to fuse cell membranes that are brought into close apposition (nanometers). PEG of low molecular weight (0.2–7 kDa) causes an osmotic imbalance across the plasmalemma that may alter membrane curvature and induce mechanical stress (Hermann et al., 1985; Money, 1989; reviewed in Malinin et al., 2002). However, this effect of PEG has only been investigated in the context of artificial vesicle systems (Mahendra et al., 2019), not *in vitro* or *in vivo*. Solutions of 1–5 kDa PEG have been used for decades to generate cell hybrids *in vitro* (Anne and Peberdy, 1976; Yang and Shen, 2006) and to repair transected axons *in vitro* (Bittner et al., 1986; Krause and Bittner, 1990; Lore et al., 1999) and *in vivo* (reviewed in Bittner et al., 2016a; Bittner et al., 2016b; Van Nest et al., 2021; Bittner et al., 2022). More recently, PEG has been used as part of a therapeutic strategy to repair transected axons in clinical settings (Bamba et al., 2016; Bittner et al., 2022; Lopez et al., 2023).

PEG-fusion repair of singly-cut or crushed ends of earthworm giant axons (Krause and Bittner, 1990), rat sciatic and spinal axons (Lore et al., 1999) rapidly (ms to seconds) restores conduction of action potentials across transection sites and morphological continuity. PEG-fusion rapidly repairs severed open ends (presumed to be vesicle-free *in vivo*) of mammalian PNS axons brought into contact by neurorrhaphy and immediately restores involuntary muscle contractions. PEG-fused nerves exhibit return of voluntary behaviors after 2–6 weeks by inducing rewiring of PNS and CNS synaptic connections (Bittner G. et al., 2000; Bittner et al., 2016a; Bittner et al., 2016b; Ghergherehchi et al., 2019).

In the absence of PEG-fusion, PEG also has a neuroprotective effect on severed CNS axons (Liu-Snyder et al., 2007), perhaps by sealing off cut ends (PEG-sealing). In this model, application of PEG to the proximal or distal stump of a transected axon causes the rapid collapse and fusion of the axolemmal leaflets, similar to the previously assumed mechanism of axonal repair depicted in Figure 1B. That is, as indicated in biochemical and dye diffusion studies, PEG-sealing bypasses all substances and sealing pathways described in Section 6 (Spaeth et al., 2012c). PEG is able to seal severed axons in both calcium-free and calcium-containing solutions and thus almost-certainly does not involve the formation of intracellular vesicles. Substances that are known to enhance (e.g., taxol) or inhibit (e.g., NEM, methylene blue) vesicle-mediated sealing had no measurable effect on PEG-sealing in B104 cells. Furthermore, the probability of successful PEG-sealing increases sigmoidally as the concentration of PEG utilized increases from 0–10 mM, indicating that PEG directly induces sealing (Spaeth et al., 2012b).

## 10 Conclusions and future directions

Eukaryotic cells commonly experience plasmalemmal damage. For decades prior to 1994, the cellular/molecular mechanisms that repair plasmalemmal injuries were not discussed or were misinterpreted. Such undocumented theories of membrane repair included lipid spreading, plasmalemmal collapse and fusion, and the formation of membrane partitions. Since 1994, experimental studies consistently show that small or large plasmalemmal lesions seal by accumulation and aggregation of vesicles and/or other membranous structures. A combination of electrophysiological measures, fluorescent dye with series confocal Z-sections, and high-resolution EM images of the injury site are necessary to accurately determine the morphological and functional state of a plasmalemmal seal. Vesicles and membrane-bound structures interact with the plasmalemma and each other to produce a complete seal by forming (1) a vesicular plug consisting of many vesicles that gradually seal the membrane disruption or (2) a patch consisting of a large wound vesicle that seals the membrane disruption in a single step. Both plug and patch sealing mechanisms assume that a set of parallel biochemical pathways and substances are primarily activated by the rapid influx of Ca^2+^ through the lesion site -- and perhaps secondarily by release of Ca^2+^ from intracellular structures (SER, mitochondria, etc.). These parallel biochemical pathways initiate less well-defined cellular events that facilitate membrane fusion and repair.

### 10.1 Cellular correlates of natural membrane sealing

Although many publications cited above provide detail on the biochemical pathways of plasmalemmal repair, few studies correlate subcellular morphological/biophysical events directly associated with biochemical substances. For instance, very little is known regarding the transport of wound-induced vesicles in cells. A “tether” that anchors vesicles to the plasmalemma has been described in crayfish MGAs (Eddleman et al., 1998a) perhaps pointing to delivery along cytoskeletal structures. Furthermore, MTs are disassembled and subsequently reassembled towards the injury site, perhaps to direct intracellular vesicle transport (Togo et al., 2000). High-resolution imaging of cells treated with various inhibitors of cytoskeletal filament polymerization and active transport are needed to evaluate the transport and aggregation of vesicles. Additionally, the role of fusogenic proteins in the repair process is not fully understood. While synaptic and Golgi fusion proteins are certainly involved in the repair process, the organization and overall contribution of these fusion complexes to vesicle aggregation is largely unknown.

Importantly, recent work has been successful in relating biochemical events with their cellular correlates. One example is the role of AnxA5 in wound stabilization. Annexins have long been known to interact with negatively charges phospholipids (such as PS) in a Ca^2+^-dependent manner (Gerke and Moss, 2002). However, it was only when high-resolution fluorescence imaging was performed on mouse perivascular cell culture that the cellular effect of AnxA5 was discovered. AnxA5 associates with PS-rich domains that surround the site of injury, forming rigid 2D crystals of AnxA5 trimers. AnxA5 arrays act to prevent expansion of the membrane injury and facilitate repair (Bouter et al., 2011). This discovery adds significant detail to our understanding of the repair process and demonstrates the importance of correlating the biochemical results to direct observations of the cellular biophysical/morphological events.

### 10.2 Clinical applications of natural membrane sealing

A better understanding of both natural mechanisms of plasmalemmal repair are almost-certainly critical to the development of new clinical treatments for conditions that involve damage to nerve cell membranes such as traumatic nerve injury, neurodegenerative diseases, muscular dystrophies, stroke and other ischemic conditions. Many neurodegenerative diseases are characterized by damage to cell membranes (reviewed in Dias and Nylandsted, 2021; Coumans et al., 2001). Muscle cells routinely experience membrane trauma (e.g., stretch injury) throughout their normal physiology. Plasma membrane instability characterizes many muscular dystrophies, brought about by mutations that compromise membrane repair and/or produce cells with fragile membranes (Davies and Nowak, 2006; Michailowsky et al., 2019). Dysregulations in annexin-family proteins, calpains, MG53, and dysferlin are involved in the development of muscular dystrophies (for review, see Croissant et al., 2021).

Mechanisms of the natural repair response may also have applications in the treatment of various cancers (Demonbreun and McNally, 2016). For example, Myoferlin-depleted Lewis Lung Carcinoma cells show a decrease in both migration and proliferation (Leung et al., 2013). Upon laser wounding, cells also show significant deficiencies in membrane repair capabilities. Myoferlin regulates endocytotic recycling/trafficking and is upregulated upon membrane injury, similar to dysferlin (Doherty et al., 2008; Demonbreun et al., 2010). Selective downregulation of ferlin-family member proteins may provide new avenues for novel cancer treatment.

### 10.3 Cellular correlates of artificial membrane sealing

PEG rapidly seals plasmalemmal lesions in milliseconds to seconds both *in vitro* and *in vivo* (Spaeth et al., 2012b; Vargas and Bittner, 2019). PEG bypasses all known substances involved in plasmalemmal repair studied to date (See Figure 3) to directly seal the lesion in a process, not yet directly observed, that is assumed to closely resemble the previously assumed mechanism of plasmalemmal collapse and fusion (See Section 4; Figure 1B). PEG might cause transected axonal leaflets to come together, fuse and seal the cut end within milliseconds to seconds, as compared with the natural methods of sealing that occur over seconds to minutes.

PEG-sealing has only been assessed by prevention of dye uptake and should now be studied more in depth using a combination of the methods discussed in this review. Individually transected crayfish MGAs can serve as a model system for studying PEG-sealing. MGAs have a large enough diameter to facilitate confocal microscopy and electrophysiological measurement. Fluorescent dye combinations allow one to observe 1) the membrane dynamics (i.e., collapse and fusion), 2) the presence or absence of intracellular vesicles, and 3) the assessment of a seal by dye exclusion. These properties will allow for study of the cellular dynamics upon PEG addition. Additionally, many of the inhibitors of substances within the natural repair biochemical pathway have been used previously in crayfish MGAs (see Section 6) allowing one to study the dependency of PEG-sealing on elements such as the cytoskeleton, wound state, etc.

### 10.4 Clinical applications of artificial membrane sealing

PEG-sealing of may provide new avenues for the treatment of locally damaged cells and aid in the improvement of behavioral recovery following PEG-fusion of peripheral nerve injuries. Ischemic conditions (e.g., stroke) that are characterized by oxygen glucose deprivation in the cell often result in plasmalemmal damage and the formation of membrane blebs (Nishimura and Lemasters, 2001; Garcia-Dorado et al., 2012). Repair of ischemic cells by the natural repair response can be insufficient to prevent cell death and/or degeneration of anucleate cytoplasmic extensions. The rapid sealing of holes and/or complete transections *via* local application of PEG could prevent apoptosis and improve behavioral recovery.
