# Supplementary material for: Repair of traumatic lesions to the plasmalemma of neurons and other cells: Commonalities, conflicts, and controversies
Source: Front Physiol. 2023 Mar 15;14:1114779. doi: 10.3389/fphys.2023.1114779 (PMC10050709; doi:10.3389/fphys.2023.1114779)
Supplement: Supplementary file 1 [file DataSheet1.docx]

**Supplemental Information**

Below is a list of numerically-labeled references to be used in conjunction with Tables 1 and 2. Numbers in front of each reference corresponds to those listed in each cell of the tables.

1. Horn A, Van der Meulen JH, Defour A, Hogarth M, Sreetama SC, Reed A, Scheffer L, Chandel NS, Jaiswal JK. Mitochondrial redox signaling enables repair of injured skeletal muscle cells. Sci Signal. 2017 Sep 5;10(495):eaaj1978. doi: 10.1126/scisignal.aaj1978. PMID: 28874604; PMCID: PMC5949579.
2. Cai C, Masumiya H, Weisleder N, Matsuda N, Nishi M, Hwang M, Ko JK, Lin P, Thornton A, Zhao X, Pan Z, Komazaki S, Brotto M, Takeshima H, Ma J. MG53 nucleates assembly of cell membrane repair machinery. Nat Cell Biol. 2009 Jan;11(1):56-64. doi: 10.1038/ncb1812. Epub 2008 Nov 30. PMID: 19043407; PMCID: PMC2990407.
3. Cai C, Weisleder N, Ko JK, Komazaki S, Sunada Y, Nishi M, Takeshima H, Ma J. Membrane repair defects in muscular dystrophy are linked to altered interaction between MG53, caveolin-3, and dysferlin. J Biol Chem. 2009 Jun 5;284(23):15894-902. doi: 10.1074/jbc.M109.009589. Epub 2009 Apr 20. PMID: 19380584; PMCID: PMC2708885.
4. Calise S, Blescia S, Cencetti F, Bernacchioni C, Donati C, Bruni P. Sphingosine 1-phosphate stimulates proliferation and migration of satellite cells: role of S1P receptors. Biochim Biophys Acta. 2012 Feb;1823(2):439-50. doi: 10.1016/j.bbamcr.2011.11.016. Epub 2011 Dec 8. PMID: 22178384.
5. Demonbreun AR, Quattrocelli M, Barefield DY, Allen MV, Swanson KE, McNally EM. An actin-dependent annexin complex mediates plasma membrane repair in muscle. J Cell Biol. 2016 Jun 20;213(6):705-18. doi: 10.1083/jcb.201512022. Epub 2016 Jun 13. PMID: 27298325; PMCID: PMC4915191.
6. Houang EM, Sham YY, Bates FS, Metzger JM. Muscle membrane integrity in Duchenne muscular dystrophy: recent advances in copolymer-based muscle membrane stabilizers. Skelet Muscle. 2018 Oct 10;8(1):31. doi: 10.1186/s13395-018-0177-7. PMID: 30305165; PMCID: PMC6180502.
7. Scheffer LL, Sreetama SC, Sharma N, Medikayala S, Brown KJ, Defour A, Jaiswal JK. Mechanism of Ca²⁺-triggered ESCRT assembly and regulation of cell membrane repair. Nat Commun. 2014 Dec 23;5:5646. doi: 10.1038/ncomms6646. PMID: 25534348; PMCID: PMC4333728.
8. Carmeille R, Bouvet F, Tan S, Croissant C, Gounou C, Mamchaoui K, Mouly V, Brisson AR, Bouter A. Membrane repair of human skeletal muscle cells requires Annexin-A5. Biochim Biophys Acta. 2016 Sep;1863(9):2267-79. doi: 10.1016/j.bbamcr.2016.06.003. Epub 2016 Jun 7. PMID: 27286750.
9. Huang Y, Laval SH, van Remoortere A, Baudier J, Benaud C, Anderson LV, Straub V, Deelder A, Frants RR, den Dunnen JT, Bushby K, van der Maarel SM. AHNAK, a novel component of the dysferlin protein complex, redistributes to the cytoplasm with dysferlin during skeletal muscle regeneration. FASEB J. 2007 Mar;21(3):732-42. doi: 10.1096/fj.06-6628com. Epub 2006 Dec 21. PMID: 17185750.
10. Huang Y, de Morrée A, van Remoortere A, Bushby K, Frants RR, den Dunnen JT, van der Maarel SM. Calpain 3 is a modulator of the dysferlin protein complex in skeletal muscle. Hum Mol Genet. 2008 Jun 15;17(12):1855-66. doi: 10.1093/hmg/ddn081. Epub 2008 Mar 11. PMID: 18334579; PMCID: PMC2900895.
11. Lek A, Evesson FJ, Lemckert FA, Redpath GM, Lueders AK, Turnbull L, Whitchurch CB, North KN, Cooper ST. Calpains, cleaved mini-dysferlinC72, and L-type channels underpin calcium-dependent muscle membrane repair. J Neurosci. 2013 Mar 20;33(12):5085-94. doi: 10.1523/JNEUROSCI.3560-12.2013. PMID: 23516275; PMCID: PMC6704986.
12. Redpath GM, Woolger N, Piper AK, Lemckert FA, Lek A, Greer PA, North KN, Cooper ST. Calpain cleavage within dysferlin exon 40a releases a synaptotagmin-like module for membrane repair. Mol Biol Cell. 2014 Oct 1;25(19):3037-48. doi: 10.1091/mbc.E14-04-0947. Epub 2014 Aug 20. PMID: 25143396; PMCID: PMC4230592.
13. Waddell LB, Lemckert FA, Zheng XF, Tran J, Evesson FJ, Hawkes JM, Lek A, Street NE, Lin P, Clarke NF, Landstrom AP, Ackerman MJ, Weisleder N, Ma J, North KN, Cooper ST. Dysferlin, annexin A1, and mitsugumin 53 are upregulated in muscular dystrophy and localize to longitudinal tubules of the T-system with stretch. J Neuropathol Exp Neurol. 2011 Apr;70(4):302-13. doi: 10.1097/NEN.0b013e31821350b0. PMID: 21412170; PMCID: PMC6309232.
14. Deak M, Clifton AD, Lucocq LM, Alessi DR. Mitogen- and stress-activated protein kinase-1 (MSK1) is directly activated by MAPK and SAPK2/p38, and may mediate activation of CREB. EMBO J. 1998 Aug 3;17(15):4426-41. doi: 10.1093/emboj/17.15.4426. PMID: 9687510; PMCID: PMC1170775.
15. Grembowicz KP, Sprague D, McNeil PL. Temporary disruption of the plasma membrane is required for c-fos expression in response to mechanical stress. Mol Biol Cell. 1999 Apr;10(4):1247-57. doi: 10.1091/mbc.10.4.1247. PMID: 10198070; PMCID: PMC25264.
16. Jimenez AJ, Maiuri P, Lafaurie-Janvore J, Divoux S, Piel M, Perez F. ESCRT machinery is required for plasma membrane repair. Science. 2014 Feb 28;343(6174):1247136. doi: 10.1126/science.1247136. Epub 2014 Jan 30. PMID: 24482116.
17. Togo T. Cell membrane disruption stimulates NO/PKG signaling and potentiates cell membrane repair in neighboring cells. *PLoS One*. 2012;7(8):e42885. doi:10.1371/journal.pone.0042885
18. Togo T. Cell membrane disruption stimulates cAMP and Ca^2+^ signaling to potentiate cell membrane resealing in neighboring cells. Biol Open. 2017 Dec 15;6(12):1814-1819. doi: 10.1242/bio.028977. PMID: 29092813; PMCID: PMC5769656.
19. Togo T, Alderton JM, Steinhardt RA. Long-term potentiation of exocytosis and cell membrane repair in fibroblasts. Mol Biol Cell. 2003 Jan;14(1):93-106. doi: 10.1091/mbc.e02-01-0056. PMID: 12529429; PMCID: PMC140230.
20. Idone V, Tam C, Goss JW, Toomre D, Pypaert M, Andrews NW. Repair of injured plasma membrane by rapid Ca2+-dependent endocytosis. J Cell Biol. 2008 Mar 10;180(5):905-14. doi: 10.1083/jcb.200708010. Epub 2008 Mar 3. PMID: 18316410; PMCID: PMC2265401.
21. Tam C, Idone V, Devlin C, Fernandes MC, Flannery A, He X, Schuchman E, Tabas I, Andrews NW. Exocytosis of acid sphingomyelinase by wounded cells promotes endocytosis and plasma membrane repair. J Cell Biol. 2010 Jun 14;189(6):1027-38. doi: 10.1083/jcb.201003053. Epub 2010 Jun 7. PMID: 20530211; PMCID: PMC2886342.
22. Benaud C, Gentil BJ, Assard N, Court M, Garin J, Delphin C, Baudier J. AHNAK interaction with the annexin 2/S100A10 complex regulates cell membrane cytoarchitecture. J Cell Biol. 2004 Jan 5;164(1):133-44. doi: 10.1083/jcb.200307098. Epub 2003 Dec 29. PMID: 14699089; PMCID: PMC2171952.
23. Boyer L, Doye A, Rolando M, Flatau G, Munro P, Gounon P, Clément R, Pulcini C, Popoff MR, Mettouchi A, Landraud L, Dussurget O, Lemichez E. Induction of transient macroapertures in endothelial cells through RhoA inhibition by Staphylococcus aureus factors. J Cell Biol. 2006 Jun 5;173(5):809-19. doi: 10.1083/jcb.200509009. PMID: 16754962; PMCID: PMC2063895.
24. Li R, Blanchette-Mackie EJ, Ladisch S. Induction of endocytic vesicles by exogenous C(6)-ceramide. J Biol Chem. 1999 Jul 23;274(30):21121-7. doi: 10.1074/jbc.274.30.21121. PMID: 10409665.
25. Togo T. Disruption of the plasma membrane stimulates rearrangement of microtubules and lipid traffic toward the wound site. J Cell Sci. 2006 Jul 1;119(Pt 13):2780-6. doi: 10.1242/jcs.03006. Epub 2006 Jun 13. PMID: 16772335.
26. Bouter, A., Gounou, C., Bérat, R. *et al.* Annexin-A5 assembled into two-dimensional arrays promotes cell membrane repair. *Nat Commun* **2,**270 (2011). https://doi.org/10.1038/ncomms1270
27. Maxwell KN, Zhou Y, Hancock JF. Rac1 Nanoscale Organization on the Plasma Membrane Is Driven by Lipid Binding Specificity Encoded in the Membrane Anchor. Mol Cell Biol. 2018 Aug 28;38(18):e00186-18. doi: 10.1128/MCB.00186-18. PMID: 29967243; PMCID: PMC6113602.
28. Babiychuk EB, Monastyrskaya K, Potez S, Draeger A. Blebbing confers resistance against cell lysis. Cell Death Differ. 2011 Jan;18(1):80-9. doi: 10.1038/cdd.2010.81. Epub 2010 Jul 2. PMID: 20596076; PMCID: PMC3131879.
29. Borgonovo B, Cocucci E, Racchetti G, Podini P, Bachi A, Meldolesi J. Regulated exocytosis: a novel, widely expressed system. Nat Cell Biol. 2002 Dec;4(12):955-62. doi: 10.1038/ncb888. PMID: 12447386.
30. Cerny J, Feng Y, Yu A, Miyake K, Borgonovo B, Klumperman J, Meldolesi J, McNeil PL, Kirchhausen T. The small chemical vacuolin-1 inhibits Ca(2+)-dependent lysosomal exocytosis but not cell resealing. EMBO Rep. 2004 Sep;5(9):883-8. doi: 10.1038/sj.embor.7400243. Erratum in: EMBO Rep. 2005 Sep;6(9):898. PMID: 15332114; PMCID: PMC1299144.
31. De Seranno S, Benaud C, Assard N, Khediri S, Gerke V, Baudier J, Delphin C. Identification of an AHNAK binding motif specific for the Annexin2/S100A10 tetramer. J Biol Chem. 2006 Nov 17;281(46):35030-8. doi: 10.1074/jbc.M606545200. Epub 2006 Sep 19. PMID: 16984913.
32. Fiedler K, Lafont F, Parton RG, Simons K. Annexin XIIIb: a novel epithelial specific annexin is implicated in vesicular traffic to the apical plasma membrane. J Cell Biol. 1995 Mar;128(6):1043-53. doi: 10.1083/jcb.128.6.1043. PMID: 7896870; PMCID: PMC2120424.
33. Chen X, Leow RS, Hu Y, Wan JM, Yu AC. Single-site sonoporation disrupts actin cytoskeleton organization. J R Soc Interface. 2014 Mar 26;11(95):20140071. doi: 10.1098/rsif.2014.0071. PMID: 24671936; PMCID: PMC4006247.
34. Cocucci E, Racchetti G, Rupnik M, Meldolesi J. The regulated exocytosis of enlargeosomes is mediated by a SNARE machinery that includes VAMP4. J Cell Sci. 2008 Sep 15;121(Pt 18):2983-91. doi: 10.1242/jcs.032029. Epub 2008 Aug 19. PMID: 18713833.
35. Sarasa-Renedo A, Tunç-Civelek V, Chiquet M. Role of RhoA/ROCK-dependent actin contractility in the induction of tenascin-C by cyclic tensile strain. Exp Cell Res. 2006 May 1;312(8):1361-70. doi: 10.1016/j.yexcr.2005.12.025. Epub 2006 Jan 30. PMID: 16448650.
36. Sinha B, Köster D, Ruez R, Gonnord P, Bastiani M, Abankwa D, Stan RV, Butler-Browne G, Vedie B, Johannes L, Morone N, Parton RG, Raposo G, Sens P, Lamaze C, Nassoy P. Cells respond to mechanical stress by rapid disassembly of caveolae. Cell. 2011 Feb 4;144(3):402-13. doi: 10.1016/j.cell.2010.12.031. PMID: 21295700; PMCID: PMC3042189.
37. Ahmed SM, Rzigalinski BA, Willoughby KA, Sitterding HA, Ellis EF. Stretch-induced injury alters mitochondrial membrane potential and cellular ATP in cultured astrocytes and neurons. J Neurochem. 2000 May;74(5):1951-60. PMID: 10800938.
38. Ciani E, Guidi S, Bartesaghi R, Contestabile A. Nitric oxide regulates cGMP-dependent cAMP-responsive element binding protein phosphorylation and Bcl-2 expression in cerebellar neurons: implication for a survival role of nitric oxide. J Neurochem. 2002 Sep;82(5):1282-9. doi: 10.1046/j.1471-4159.2002.01080.x. PMID: 12358775.
39. Hervera A, De Virgiliis F, Palmisano I, Zhou L, Tantardini E, Kong G, Hutson T, Danzi MC, Perry RB, Santos CXC, Kapustin AN, Fleck RA, Del Río JA, Carroll T, Lemmon V, Bixby JL, Shah AM, Fainzilber M, Di Giovanni S. Reactive oxygen species regulate axonal regeneration through the release of exosomal NADPH oxidase 2 complexes into injured axons. Nat Cell Biol. 2018 Mar;20(3):307-319. doi: 10.1038/s41556-018-0039-x. Epub 2018 Feb 12. Erratum in: Nat Cell Biol. 2018 Mar 8;: PMID: 29434374.
40. Spaeth CS, Robison T, Fan JD, Bittner GD. Cellular mechanisms of plasmalemmal sealing and axonal repair by polyethylene glycol and methylene blue. J Neurosci Res. 2012 May;90(5):955-66. doi: 10.1002/jnr.23022. Epub 2012 Feb 3. PMID: 22302626.
41. Spaeth CS, Spaeth EB, Wilcott RW, Fan JD, Robison T, Bittner GD. Pathways for plasmalemmal repair mediated by PKA, Epac, and cytosolic oxidation in rat B104 cells in vitro and rat sciatic axons ex vivo. Dev Neurobiol. 2012 Nov;72(11):1399-414. doi: 10.1002/dneu.20998. Epub 2012 Jul 13. PMID: 22076955.
42. Spaeth CS, Fan JD, Spaeth EB, Robison T, Wilcott RW, Bittner GD. Neurite transection produces cytosolic oxidation, which enhances plasmalemmal repair. J Neurosci Res. 2012 May;90(5):945-54. doi: 10.1002/jnr.22823. PMID: 22497022.
43. Eddleman CS, Bittner GD, Fishman HM. Barrier permeability at cut axonal ends progressively decreases until an ionic seal is formed. Biophys J. 2000 Oct;79(4):1883-90. doi: 10.1016/S0006-3495(00)76438-1. PMID: 11023894; PMCID: PMC1301080.
44. C.S. Eddleman, G.D. Bittner, and H.M. Fishman. 2003. SEM comparison of severed ends of giant axons isolated from squid (*Loligo pealei*) and crayfish (*Procambarus clarkii*). Biol Bull. 203: 219 – 220. PubMed PMID: 12414587.
45. Gallant PE, Hammar K, Reese TS. Cytoplasmic constriction and vesiculation after axotomy in the squid giant axon. J Neurocytol. 1995 Dec;24(12):943-54. doi: 10.1007/BF01215644. PMID: 8719821.
46. Riske KA, Dimova R. Electro-deformation and poration of giant vesicles viewed with high temporal resolution. Biophys J. 2005 Feb;88(2):1143-55. doi: 10.1529/biophysj.104.050310. Epub 2004 Dec 13. PMID: 15596488; PMCID: PMC1305119.
47. Corrotte M, Almeida PE, Tam C, Castro-Gomes T, Fernandes MC, Millis BA, Cortez M, Miller H, Song W, Maugel TK, Andrews NW. Caveolae internalization repairs wounded cells and muscle fibers. Elife. 2013 Sep 17;2:e00926. doi: 10.7554/eLife.00926. PMID: 24052812; PMCID: PMC3776555.
48. Vaughan EM, You JS, Elsie Yu HY, Lasek A, Vitale N, Hornberger TA, Bement WM. Lipid domain-dependent regulation of single-cell wound repair. Mol Biol Cell. 2014 Jun 15;25(12):1867-76. doi: 10.1091/mbc.E14-03-0839. Epub 2014 Apr 30. PMID: 24790096; PMCID: PMC4055266.
49. Benink HA, Bement WM. Concentric zones of active RhoA and Cdc42 around single cell wounds. J Cell Biol. 2005 Jan 31;168(3):429-39. doi: 10.1083/jcb.200411109. PMID: 15684032; PMCID: PMC2171735.
50. Davenport NR, Sonnemann KJ, Eliceiri KW, Bement WM. Membrane dynamics during cellular wound repair. Mol Biol Cell. 2016 Jul 15;27(14):2272-85. doi: 10.1091/mbc.E16-04-0223. Epub 2016 May 25. PMID: 27226483; PMCID: PMC4945144.
51. Todora MA, Fishman HM, Krause TL, Bittner GD. Shortening of a severed squid giant axon is non-uniform and occurs in two phases. Neurosci Lett. 1994 Sep 26;179(1-2):57-9. doi: 10.1016/0304-3940(94)90934-2. PMID: 7845625.
52. Krause TL, Fishman HM, Ballinger ML, Bittner GD. Extent and mechanism of sealing in transected giant axons of squid and earthworms. J Neurosci. 1994 Nov;14(11 Pt 1):6638-51. doi: 10.1523/JNEUROSCI.14-11-06638.1994. PMID: 7965066; PMCID: PMC6577266.
53. Fishman HM, Krause TL, Miller AL, Bittner GD. Retardation of the spread of extracellular Ca2+ into transected, unsealed squid giant axons. Biol Bull. 1995 Oct-Nov;189(2):208-9. doi: 10.1086/BBLv189n2p208. PMID: 8541404.
54. Eddleman, C. S., Ballinger, M. L., Smyers, M. E., Godell, C. M., Fishman, H. M., and Bittner, G. D. (1997). Repair of Plasmalemmal Lesions by Vesicles. *Proceedings of the National Academy of Sciences of the United States of America*, *94*(9), 4745–4750. http://www.jstor.org/stable/42083
55. Godell CM, Smyers ME, Eddleman CS, Ballinger ML, Fishman HM, Bittner GD. Calpain activity promotes the sealing of severed giant axons. Proc Natl Acad Sci U S A. 1997 Apr 29;94(9):4751-6. doi: 10.1073/pnas.94.9.4751. PMID: 9114063; PMCID: PMC20796.
56. Ballinger ML, Blanchette AR, Krause TL, Smyers ME, Fishman HM, Bittner GD. Delaminating myelin membranes help seal the cut ends of severed earthworm giant axons. J Neurobiol. 1997 Dec;33(7):945-60. doi: 10.1002/(sici)1097-4695(199712)33:7<945::aid-neu6>3.0.co;2-8. PMID: 9407015.
57. Eddleman CS, Ballinger ML, Smyers ME, Fishman HM, Bittner GD. Endocytotic formation of vesicles and other membranous structures induced by Ca2+ and axolemmal injury. J Neurosci. 1998 Jun 1;18(11):4029-41. doi: 10.1523/JNEUROSCI.18-11-04029.1998. PMID: 9592084; PMCID: PMC6792792.
58. Lichstein, J.W., Ballinger, M.L., Blanchette, A.R., Fishman, H.M. and Bittner, G.D. (2000), Structural changes at cut ends of earthworm giant axons in the interval between dye barrier formation and neuritic outgrowth. J. Comp. Neurol., 416: 143-157. [https://doi.org/10.1002/(SICI)1096-9861(20000110)416:2<143::AID-CNE2>3.0.CO;2-3](https://doi.org/10.1002/(SICI)1096-9861(20000110)416:2%3C143::AID-CNE2%3E3.0.CO;2-3)
59. Detrait E, Eddleman CS, Yoo S, Fukuda M, Nguyen MP, Bittner GD, Fishman HM. Axolemmal repair requires proteins that mediate synaptic vesicle fusion. J Neurobiol. 2000 Sep 15;44(4):382-91. doi: 10.1002/1097-4695(20000915)44:4<382::aid-neu2>3.0.co;2-q. PMID: 10945894.
60. Detrait ER, Yoo S, Eddleman CS, Fukuda M, Bittner GD, Fishman HM. Plasmalemmal repair of severed neurites of PC12 cells requires Ca(2+) and synaptotagmin. J Neurosci Res. 2000 Nov 15;62(4):566-73. doi: 10.1002/1097-4547(20001115)62:4<566::AID-JNR11>3.0.CO;2-4. PMID: 11070500.
61. Yoo, S., Nguyen, M.P., Fukuda, M., Bittner, G.D., and Fishman, H.M. (2003). Plasmalemmal sealing of transected mammalian neurites is a gradual process mediated by Ca2+‐regulated proteins. *Journal of Neuroscience Research, 74*.
62. Yoo S, Bottenstein JE, Bittner GD, Fishman HM. Survival of mammalian B104 cells following neurite transection at different locations depends on somal Ca2+ concentration. J Neurobiol. 2004 Aug;60(2):137-53. doi: 10.1002/neu.20005. PMID: 15266646.
63. Zuzek A, Fan JD, Spaeth CS, Bittner GD. Sealing of transected neurites of rat B104 cells requires a diacylglycerol PKC-dependent pathway and a PKA-dependent pathway. Cell Mol Neurobiol. 2013 Jan;33(1):31-46. doi: 10.1007/s10571-012-9868-5. Epub 2012 Aug 3. PMID: 22865002.
64. Terasaki M, Miyake K, McNeil PL. Large plasma membrane disruptions are rapidly resealed by Ca2+-dependent vesicle-vesicle fusion events. J Cell Biol. 1997 Oct 6;139(1):63-74. doi: 10.1083/jcb.139.1.63. PMID: 9314529; PMCID: PMC2139822.
65. McNeil PL, Baker MM. Cell surface events during resealing visualized by scanning-electron microscopy. Cell Tissue Res. 2001 Apr;304(1):141-6. doi: 10.1007/s004410000286. PMID: 11383880.
66. Mellgren RL, Zhang W, Miyake K, McNeil PL. Calpain is required for the rapid, calcium-dependent repair of wounded plasma membrane. J Biol Chem. 2007 Jan 26;282(4):2567-75. doi: 10.1074/jbc.M604560200. Epub 2006 Nov 22. PMID: 17121849.
67. Steinhardt RA, Bi G, Alderton JM. Cell membrane resealing by a vesicular mechanism similar to neurotransmitter release. Science. 1994 Jan 21;263(5145):390-3. doi: 10.1126/science.7904084. PMID: 7904084.
